# Multiplicative versus additive modelling of causal effects using instrumental variables for survival outcomes – a comparison

**DOI:** 10.1177/09622802241293765

**Published:** 2024-12-10

**Authors:** Eleanor R John, Michael J Crowther, Vanessa Didelez, Nuala A Sheehan

**Affiliations:** 1 Department of Health Sciences, 4488 University of Leicester, UK; 2 Red Door Analytics, Stockholm, Sweden; 3 Leibniz Institute for Prevention Research and Epidemiology – BIPS, Berlin, Germany; 4 Faculty of Mathematics and Computer Science, University of Bremen, Germany

**Keywords:** Causal effects, instrumental variables, time-to-event outcomes

## Abstract

Instrumental variables (IVs) methods have recently gained popularity since, under certain assumptions, they may yield consistent causal effect estimators in the presence of unmeasured confounding. Existing simulation studies that evaluate the performance of IV approaches for time-to-event outcomes tend to consider either an additive or a multiplicative data-generating mechanism (DGM) and have been limited to an exponential constant baseline hazard model. In particular, the relative merits of additive versus multiplicative IV models have not been fully explored. All IV methods produce less biased estimators than naïve estimators that ignore unmeasured confounding, unless the IV is very weak and there is very little unmeasured confounding. However, the mean squared error of IV estimators may be higher than that of the naïve, biased but more stable estimators, especially when the IV is weak, the sample size is small to moderate, and the unmeasured confounding is strong. In addition, the sensitivity of IV methods to departures from their assumed DGMs differ substantially. Additive IV methods yield clearly biased effect estimators under a multiplicative DGM whereas multiplicative approaches appear less sensitive. All can be extremely variable. We would recommend that survival probabilities should always be reported alongside the relevant hazard contrasts as these can be more reliable and circumvent some of the known issues with causal interpretation of hazard contrasts. In summary, both additive IV and Cox IV methods can perform well in some circumstances but an awareness of their limitations is required in analyses of real data where the true underlying DGM is unknown.

## 1. Introduction

Randomised controlled trials (RCTs) are getting shorter and more streamlined as regulatory bodies, such as the Food and Drug Administration and European Medicines Agency, look to speed up access to innovative health care. Policy makers need to conduct heath technology assessments to determine whether treatments are actually cost-effective before approving them for clinical practice. The increasing availability of very large data resources, such as electronic health record (EHR) data, means that we now have vast amounts of observational data that include information on outcomes that are not always possible to evaluate in a randomised trial. For example, most trials do not run for long enough to observe data on overall survival and use progression-free survival as a surrogate endpoint. Even though regulators and licensing bodies will approve a treatment based on its performance with regard to progression-free survival, the longer follow-up periods that are found in cancer registry or EHR data are required to support cost-effectiveness analyses. Due to the limited availability of RCT evidence, there is hence heightened interest in reliable methods for treatment effect estimation from observational data with a view to supplementing, or possibly even to replacing, evidence from RCTs.^1^ Observational effect estimators can be biased in the presence of unmeasured confounding and while there are many methods that try to alleviate such bias, their relative performance is unclear.^2^ Importantly, if we want to replace or supplement evidence from an RCT, we need methods for observational data that provide reliable and valid results under plausible assumptions.

Unlike other adjustment methods, such as adjustment based on the propensity score, instrumental variable (IV) approaches *can* consistently estimate causal effects in the presence of unmeasured confounding provided certain conditions are satisfied. For exposure X and outcome Y , let $C_{U}$ represent a set of unmeasured factors confounding the association between X and Y in the sense that $C_{U}$ would be sufficient to adjust for confounding if it were measured, that is, $P(y|C_{U},do(X=x))=P(y|C_{U},X=x)$ . Here, the conditional distribution P(Y=y|do(X=x)) represents the distribution of Y in the hypothetical situation where the whole population has had their exposure set to X=x .^3^ For a variable Z to be an IV it needs to satisfy the following three conditions (possibly conditional on additional measured covariates):

(i)
Z is associated with X ;
(ii)
Z is associated with the outcome Y only through X or, more formally, $Z\underset{\;\_}{\|}Y|X,C_{U}$ ; and
(iii)
Z is independent of unmeasured confounders $C_{U}$ .

Note that only the first of these can be verified empirically as the others involve the unmeasured confounding $C_{U}$ . Several additional strict, and often unverifiable assumptions, are required for point identification of causal effects.^4^ Although well developed for additive no-interaction models for continuous outcomes, application to binary and time-to-event outcomes is more problematic due to the non-collapsibility of the relevant effect measures.^5–7^ Approximations have been developed for binary outcomes but are not guaranteed to target causal effects.^5,8–13^

The Cox-proportional hazards model, which assumes multiplicative covariate effects on the hazard scale, is the most widely used model for time-to-event data but there has been limited development of IV methods for this model until recently.^14–17^ A two-stage approach, analogous to that used for continuous outcomes, is easy to implement and often used in practice but is not guaranteed to be consistent for a causal effect.^18^ The structural Cox model,^17^ on the other hand, targets a particular causal hazard ratio: the causal effect of treatment among treated subjects. Alternatively, additive hazards IV models have been proposed for time-to-event data since the hazard difference *is* a collapsible effect measure under the additive hazard model.^7,19–21^ In particular, the two-stage residual inclusion (2SRI) approach, also known as the control function approach, consistently estimates the marginal causal hazard difference when the exposure is binary.^7^ Both additive hazards and Cox IV methods have been applied to observational data, for example, Desai et al.^22^ used an additive control function approach to assess the effect of osteoporosis medication use on subsequent fractures whereas Guo et al.^23^ used a two-stage Cox IV model to consider the effect of body mass index (BMI) on breast cancer survival in a Mendelian randomisation (MR) study. In practice, the Cox model tends to be more widely used than the additive hazards model and remains the standard analysis model for clinical trials. This may be due to the lack of restriction on the additive hazards model to ensure a non-negative hazard and hence valid survival probabilities.^24^ Yet, if we consider the RCT as the gold standard, a method that identifies a marginal causal effect might seem preferable. It is hence important to assess the sensitivity of these additive IV models to departures from the additivity of covariate effects.

Previous simulation studies to compare IV approaches to time-to-event analyses either focus on the relative performance of Cox IV methods where the data are generated with multiplicative covariate effects, that is, under a multiplicative data-generating mechanism (DGM),^16–18^ or on the performance of additive IV methods under an additive DGM.^19,20^ Recently, Cho et al.^25^ assessed the performance of both additive and Cox IV methods but under the relevant DGM for each method. These studies also tended to focus on an exponential model for the baseline hazard which remains constant over time.

Here, we present an extensive simulation study that compares these different IV approaches in as unprejudiced a way as possible using additive-hazard and multiplicative-hazard DGMs alongside different baseline hazard models. We also consider much larger sample sizes than used in other studies reflecting the large samples that we would expect from EHR data. In addition to targeting causal contrasts on different scales, for example, hazard differences versus hazard ratios, some of the methods target conditional, or sub-group specific, rather than marginal, or population, parameters. While our primary interest is in the consistent estimation of a marginal causal parameter comparing treatment versus control, as targeted by an RCT, we also consider how each estimator performs with regard to its target estimand on the relevant scale, regardless of the DGM. The additive IV methods are always assessed with regard to how well they recover the relevant true hazard difference and the Cox IV method estimates are always compared with the relevant true hazard ratio. Despite their fundamental differences, all these models target survival probability predictions so we also consider survival probabilities to compare the different models.

The paper is structured as follows. The first section describes the analysis models to be evaluated, the simulation of survival times using exponential and Weibull baseline hazard models and the design of the simulation study. We discuss how to make the different scenarios comparable for additive and multiplicative DGMs and how to obtain the true estimands for each scenario. The results of the study are then presented. We then apply the methods to a study of the effect of statin treatment on the time-to-developing Type 2 diabetes and we conclude with a discussion of our findings where we make some practical recommendations and propose potential avenues for further work.

## 2. Simulation study

In this section, we present the analysis models to be compared and their target estimands. We then describe how we simulate observational time-to-event data and how we endeavour to make the comparison between multiplicative and additive models as fair as possible over a wide range of scenarios. We then show how we evaluate the relevant estimands for each scenario and finally describe the simulation study itself.

### 2.1. Analysis methods

For survival times T , binary treatment variable X , binary instrumental variable Z , and measured covariates $C_{M}$ , effects of treatment on survival and survival probability predictions were considered from the following models:

(i)
$A_{N}$ : Naïve additive hazards regression adjusting for $C_{M}$ .
(ii)
$C_{N}$ : Naïve Cox proportional hazards regression adjusting for $C_{M}$ .
(iii) Additive hazards IV methods.
  -
$A_{2S}$ : Two-stage additive IV method.
  -
$A_{\mathrm{RI}}$ : 2SRI additive IV method.
(iv) Cox IV methods.
  -
$C_{2S}$ : Two-stage Cox IV method.
  -
$C_{\mathrm{St}}$ : Structural Cox IV method.

#### 2.1.1. Naïve additive hazards regression ( $A_{N}$ )

An adjusted additive hazards model, also known as a semi-parametric additive hazards model^26,27^:

$$h(t|X,C_{M})=h_{0}(t)+\theta_{X}^{A_{N}}X+\theta_{C_{M}}^{A_{N}}C_{M}$$

where $h_{0}(t)$ is the baseline hazard function. The parameter $\theta_{X}^{A_{N}}$ is a causal hazard difference only if there are no unmeasured covariates $C_{U}$ , that is, $C_{M}$ is sufficient to adjust for confounding, and if the model is correctly specified in that there is no effect modification by $C_{M}$ and no time dependence of covariate effects on the hazard. Note that the marginal and conditional (on $C_{M}$ ) causal hazard differences would then be identical, that is, the hazard difference is collapsible.^21,28^

#### 2.1.2. Naïve Cox proportional hazards regression ( $C_{N}$ )

An adjusted Cox proportional hazards model:

$$h(t|X,C_{M})=h_{0}(t)\exp\;\left(\theta_{0}^{C_{N}}+\theta_{X}^{C_{N}}X+\theta_{C_{M}}^{C_{N}}C_{M}\right)$$

where $h_{0}(t)$ is the baseline hazard function. The parameter $\theta_{X}^{C_{N}}$ can be seen as a conditional (on $C_{M}$ ) causal log hazard ratio only if $C_{M}$ were sufficient to adjust for confounding and if the model is correctly specified. Note that it does not equal the marginal causal log hazard ratio.

#### 2.1.3. Two-stage additive IV method ( $A_{2S}$ )

This approach is inspired by two-stage methods for linear no-interaction models. The first stage is a logistic regression of X on the IV, Z , and measured covariates $C_{M}$ :

(1) $$\log\;\left(\frac{P[X=1|Z,C_{M}]}{1-P[X=1|Z,C_{M}]}\right)=\eta_{0}+\eta_{Z}Z+\eta_{C_{M}}C_{M}$$

Predicted treatment values $\hat{X}=\hat{P}[X=1|Z,C_{M}]$ are obtained for each subject from (1) and the second stage comprises an additive hazards regression of the outcome on these predictions and observed covariates ( $C_{M}$ ):

$$h(t|X,C_{M})=h_{0}(t)+\theta_{X}^{A_{2S}}\hat{X}+\theta_{C_{M}}^{A_{2S}}C_{M}$$

While a two-stage model can provide consistent estimation under certain assumptions for continuous X , the above will be biased for the causal hazard difference when X is binary.^7^

#### 2.1.4. 2SRI additive IV method ( $A_{RI}$ )

This approach takes the estimated residuals, $\hat{\Delta }=X-\hat{P}(X=1|Z,C_{M})$ , from the first-stage regression of the instrument on the exposure (1) and substitutes them into the second-stage outcome model as an attempt to control for unmeasured confounding:

(2) $$\begin{array}{rl} h(t|X,C_{M},Z) & =h_{0}(t)+\theta_{X}^{A_{\mathrm{RI}}}X\\ & \;+\theta_{C_{M}}^{A_{\mathrm{RI}}}C_{M}\\ & \;+\left(\rho_{0}(t)+\rho_{1}(t)Z\right)\Delta \end{array}$$

for baseline hazard $h_{0}(t)$ and $\Delta =X-P(X=1|Z,C_{M})$ . The variability explained by unmeasured confounding is accounted for in the ρ parameters. Under the model assumptions, the parameter θXARIθX22 represents the (marginal) causal hazard difference and is consistently estimated by the 2SRI approach when X is binary.^7^

#### 2.1.5. Two-stage Cox IV method ( $C_{2S}$ )

This method is analogous to the two-stage additive IV method above. The first stage fits a logistic regression of X on Z , and $C_{M}$ (1) and the second stage fits a Cox proportional hazards model to the predicted values, $\hat{X}$ , from that model and $C_{M}$ :

$$h(t|X,C_{M})=h_{0}(t)\exp\;(\theta_{0}^{C_{2S}}+\theta_{X}^{C_{2S}}\hat{X}+\theta_{C_{M}}^{C_{2S}}C_{M})$$

Because of non-linearity, the treatment coefficient $\theta_{X}^{C_{2S}}$ does not actually equal a causal effect measure and, as mentioned above, the approach is likely to be biased.

#### 2.1.6. Structural Cox IV method ( $C_{St}$ )

Martinussen et al.^17^ propose an approach to estimate the causal effect of treatment on the treated based on a structural Cox model:

$$\frac{h_{T^{x}}(t|X=x,Z)}{h_{T^{0}}(t|X=x,Z)}=\exp\;(\theta_{X}^{C_{\mathrm{St}}}X)\text{ for all }x$$

where $T^{x}$ is the potential survival time had the treatment been set to x . The numerator gives the hazard in the group of exposed individuals X=x had their exposure been set to this value by intervention. The model contrasts this with the hazard in that same group of exposed individuals had their exposure been set to a reference level X=0 or, as in our case, had they not been exposed. The structural Cox model therefore gives a measure of the causal effect of treatment in the treated (ETT) which is a subgroup effect.

Under the IV assumptions and the above structural model, an estimator is obtained as a solution of

(3) $$0=\sum_{i=1}^{n}\{g(Z_{i})-E[g(Z)]\}P(T_{i}>t|X_{i},Z_{i}{)}^{\exp\;(-\theta_{X}^{C_{\mathrm{St}}}X_{i})}$$

for $\theta_{X}^{C_{\mathrm{St}}}$ using g -estimation, where g is a function of Z such that E[g(Z)]=0 (e.g. g(Z)=Z−E(Z) ). The above procedure may result in different estimates of $\theta_{X}^{C_{\mathrm{St}}}$ for different times t . The quantities $P(T_{i}>t|X_{i},Z_{i})$ need to be estimated. There are several ways to do this, including via a Cox proportional hazards model which we will use here.^17^ The model can be extended to incorporate measured covariates to yield a causal effect conditional on $C_{M}$ .

Table 1 provides a summary of the analysis methods to be compared together with the causal contrasts they target and their required assumptions.

**Table 1.** Target parameters and model assumptions for different analysis methods. All IV methods require no interaction or effect modification by Z on the relevant scale together with causal consistency.

Analysis method	Estimand	Causal*	Additional assumptions
Naïve additive	Marginal HD	Yes	All confounders measured (not true in this study).
Naïve Cox	Conditional HR	Yes	All confounders measured (not true in this study)
Additive two-stage IV	Marginal HD	Yes	Linearity of the effect of the unmeasured confounder on the hazard. Continuous exposure X (not true in this study).
Additive 2SRI IV	Marginal HD	Yes	Effect of treatment on the hazard is additive. Linearity of the effect of the unmeasured confounder on the hazard.
Cox two-stage IV	Conditional HR	No	
Structural Cox IV	ETT HR	Yes	

IV: instrumental variable; HD: hazard difference; HR; hazard ratio; 2SRI: two-stage residual inclusion; ETT: effect of treatment in the treated.

*Can be shown to consistently estimate a causal effect under the assumptions.

### 2.2. Data-generating mechanisms (DGMs)

A binary instrumental variable Z was generated from a binomial distribution with probability 1/2 and the covariates $C_{M}$ and $C_{U}$ , representing measured and unobserved confounders, respectively, were generated from a standard normal distribution:

$$\begin{array}{rl} Z & ∼\mathrm{Binom}(1,0.5)\\ C_{M} & ∼N(0,1)\\ C_{U} & ∼N(0,1) \end{array}$$

Probability of treatment P , dependent on Z , $C_{M}$ and $C_{U}$ , was generated from a probit model and binary treatment, X , derived from a binomial distribution with that probability:

(4) $$\begin{array}{rl} P_{i} & =\Phi (\alpha_{0}+\alpha_{Z}Z_{i}+\alpha_{C_{M}}{C_{M}}_{i}+\alpha_{C_{U}}{C_{U}}_{i})\\ X_{i} & ∼\mathrm{Binom}\;(1,P_{i})\;\text{for each individual}\;i \end{array}$$

where $X_{i}=1$ represents an individual in the treated group and $X_{i}=0$ is an untreated individual. We note that this first-stage model is slightly different from that assumed by the two-stage IV methods described above.

Survival times T were simulated to be dependent on X , $C_{M}$ and $C_{U}$ using both exponential and Weibull baseline hazards alongside additive and multiplicative DGMs. When the baseline hazard follows an exponential distribution, the survival functions are invertible for both additive and multiplicative DGMs and survival times can be generated using the inversion method.^29^ When the baseline hazard follows a Weibull distribution, the survival function is only invertible for a multiplicative DGM where the inversion method is applicable. For an additive DGM, numerical integration and root-finding methods proposed by Crowther et al.^30^ are required. The formulae for the survival and hazard functions for different baseline hazard functions and DGMs are given in Table 2. Linear predictors with different coefficients are specified for additive ( $W^{T}\beta_{A}$ ) and multiplicative ( $W^{T}\beta_{M}$ ) DGMs, as will be discussed below. We will use β to refer to the relevant coefficient vector $\beta_{A}$ or $\beta_{M}$ whenever this is unambiguous. For the Weibull distribution, the shape parameter γ can be altered to give an increasing or decreasing baseline hazard. For this simulation, γ was set to 1.5 and 0.5 for the increasing and decreasing Weibull hazards, respectively. We note that the multiplicative DGM above does not necessarily guarantee the assumption that there is no effect modification by Z , required for the structural Cox estimator, due to non-collapsibilty.

**Table 2.** Hazard and survival functions under different data-generating mechanisms (DGMs) and parametric distributions where γ was set to 1.5 for an increasing Weibull hazard and 0.5 for the decreasing hazard. The scale parameter, λ , assumes different values for different baseline hazard functions and DGMs.

	Hazard function	Survival function	Linear predictor
Additive	$h(t)=h_{0}(t)+W^{T}\beta_{A}$	$S(t)=S_{0}(t)\exp\;(W^{T}\beta_{A})$	$W^{T}\beta_{A}=\beta_{0}^{A}+\beta_{X}^{A}X+\beta_{C_{M}}^{A}C_{M}+\beta_{C_{U}}^{A}C_{U}$
Multiplicative	$h(t)=h_{0}(t)\exp\;(W^{T}\beta_{M})$	$S(t)=S_{0}{\left(t\right)}^{\exp\;(W^{T}\beta_{M})}$	$W^{T}\beta_{M}=\beta_{0}^{M}+\beta_{X}^{M}X+\beta_{C_{M}}^{M}C_{M}+\beta_{C_{U}}^{M}C_{U}$
Exponential	$h_{0}(t)=\lambda$	$S_{0}(t)=\exp\;(-\lambda t)$	
Weibull	$h_{0}(t)=\lambda \gamma t^{\gamma -1}$	$S_{0}(t)=\exp\;(-\lambda t^{\gamma })$	

The coefficients $\beta_{M}$ and $\beta_{A}$ for the linear predictors in Table 2 were chosen to reflect a wide range of different scenarios and to make the survival curves under the additive and multiplicative DGMs as similar as possible for each scenario. Follow-up time was five years. We considered three different magnitudes of treatment effect (small, medium and large) corresponding to three different values for the $\beta_{X}$ parameter. Four strengths of IV (very weak, weak, moderate and strong) were obtained by varying the $\alpha_{Z}$ parameter in equation (4) and three strengths of confounding (weak, moderate and strong) were generated by varying the $\alpha_{C_{U}}$ and $\beta_{C_{U}}$ parameters.

Generating similar survival curves under additive and multiplicative DGMs was a challenge, particularly so for a Weibull baseline and multiplicative DGM as the shape of the survival curves, in that case, is especially difficult to capture using additive hazard models. We started with the multiplicative DGM and then sought corresponding values for the additive DGM. The specified coefficients for each scenario under a multiplicative DGM ( $\beta_{M}$ ) are shown in the second column of Table 3. Since it was impossible to get the curves to match over the duration of follow-up, we matched on 5-year survival. Care also had to be taken with the additive DGMs in order to keep the hazard positive and survival probabilities valid (i.e. between 0 and 1 ) as there is no restriction on the model to ensure this.^24,27,31^ Also, the additive hazard function tended to increase much more quickly than the corresponding multiplicative hazard, so we restricted the maximum follow-up length in the additive case to obtain larger survival probabilities at the end of follow-up and then re-scaled the simulated times to get matching follow-up length. We consider two values of the survival probability at 5 years, high ( ∼60% ) and low ( ∼30% ), by setting the scale parameter $\lambda \equiv \lambda_{M}$ to give the desired expected survival probability in the reference group (i.e. with $W^{T}\beta_{M}=0$ ) for each of the three baseline hazard functions. The corresponding values of the scale parameter, $\lambda_{M}$ for high and low survival probabilities at the end of follow-up are shown in Table 4 for the exponential and Weibull hazards. Different coefficients, $\beta_{A}$ , shown in the third column of Table 3 and a re-scaling ( $\lambda_{A}=0.1\lambda_{M}$ ) were required to obtain matching survival proportions at the end of follow-up under an additive DGM. Further details of the process to obtain similar survival curves under additive and multiplicative DGMs are provided in Supplemental Appendixes.

**Table 3.** Coefficient values used to simulate data in the different simulation scenarios. The last column gives the term that will be used to refer to each corresponding scenario.

	Multiplicative ( $\beta_{M}$ )	Additive ( $\beta_{A}$ )	Scenario term
Treatment coefficient ( $\beta_{X}$ )	−0.1, −0.2, −0.4	−0.035, −0.07, −0.13	Corresponding effect: small, medium and large
Strength IV ( $\alpha_{Z}$ )	0.1, 0.3, 0.5, 0.8	0.1, 0.3, 0.5, 0.8	Very weak, weak, moderate and strong
Confounding $\left(\left(\begin{array}{c} \alpha_{C_{U}}\\ \beta_{C_{U}} \end{array}\right)\right)$	$\left(\begin{array}{c} -0.1\\ 0.1 \end{array}\right)$ , $\left(\begin{array}{c} -0.3\\ 0.3 \end{array}\right)$ , $\left(\begin{array}{c} -0.5\\ 0.5 \end{array}\right)$	$\left(\begin{array}{c} -0.1\\ 0.01 \end{array}\right)$ , $\left(\begin{array}{c} -0.3\\ 0.04 \end{array}\right)$ , $\left(\begin{array}{c} -0.5\\ 0.05 \end{array}\right)$	weak, moderate and strong

IV: instrumental variable. $\alpha_{0}$ =0, $\alpha_{C_{M}}=-0.2$ for all scenarios. $\beta_{0}^{M}=0$ $,\beta_{C_{M}}^{M}=0.2$ and $\beta_{0}^{A}=0.4$ , $\beta_{C_{M}}^{A}=0.02$ .

**Table 4.** Scale parameter ( $\lambda_{M}$ ) required for the two desired survival probabilities at 5-year follow-up for different baseline hazards and a multiplicative data-generating mechanism (DGM). For the additive DGM the scale parameter was taken to be $\lambda_{A}=0.1\lambda_{M}$ .

	Exponential
S(5)=0.30	0.241
S(5)=0.60	0.102
	Decreasing Weibull ( γ=0.5 )
S(5)=0.30	0.5384
S(5)=0.60	0.2284
	Increasing Weibull ( γ=1.5 )
S(5)=0.30	0.1077
S(5)=0.60	0.0457

In total, 432 different scenarios were investigated corresponding to all combinations of the three baseline hazard distributions, two 5 -year survival probabilities, three treatment effects, four IV strengths, and three confounding strengths for both additive and multiplicative DGMs.

The motivation behind trying to match survival curves is so that they reflect plausible survival proportions observed in real data examples, whatever the underlying (unknown) DGM. For example, in the clinical practice research database (CPRD) analysis, the observed survival at the end of follow-up is above 75% which is compatible with the high survival scenarios here.

The F-statistic from a linear model of the IV on treatment is commonly used as a measure of the strength of the instrument. An F-statistic over 10 is often suggested as indicative of a strong IV.^32,33^ Since treatment is binary in this simulation, and the F-statistic depends heavily on sample size, a more appropriate measure of strength of IV is proposed. The treatment probabilities reflecting different strengths of IV for the three different confounding strengths are shown in Supplemental Appendix A (Supplemental Table A1). The difference in probability of treatment between the two IV groups (those with Z=1 , compared to those with Z=0 ) increases with increasing strength of IV and ranges from about 4 percentage points for a very weak IV to about 28 percentage points for a strong IV. We note that this is not a perfect measure either as it does not account for the the number of observations.

### 2.3. Evaluating the causal estimands

As noted above (Table 1), different analysis models target different causal effects (i.e. marginal, conditional on $C_{M}$ or ETT) and the relevant effect is not necessarily identical to the coefficient of X , $\beta_{X}^{A}$ or $\beta_{X}^{M}$ , in the corresponding DGM of Table 2. While this is obvious for the multiplicative models since the hazard ratio is non-collapsible, it is also possible that the true causal hazard difference does not equal the $\beta_{X}^{A}$ coefficient used in the DGM due to the re-scaling of time when matching 5 -year survival probabilities. The target true causal effect is a contrast in terms of intervening in X to set it either to treatment do(X=1) or to control do(X=0) . It will refer to the different contrasts that we will consider, be they hazard ratios or hazard differences. For example, a marginal causal effect can be defined as follows:

Causal hazard difference:

h(t|do(X=1))−h(t|do(X=0))

Causal hazard ratio:

$$\frac{h(t|do(X=1))}{h(t|do(X=0))}$$

Causal survival probability difference:

S(t|do(X=1))−S(t|do(X=0))

with or without dependence on t .

Regardless of the underlying DGM, the true causal contrast can be evaluated in terms of a hazard ratio and hazard difference for each simulation scenario allowing for bias in the multiplicative Cox methods to be assessed under a truly additive DGM and bias in the additive hazards methods to be assessed under a truly multiplicative DGM. The actual value of the true causal contrast will depend on several factors such as the distribution of the baseline hazard, strength of confounding, treatment coefficient and whether the DGM is additive or multiplicative.

The true causal hazard ratios, hazard differences and true survival probabilities for each treatment arm could be approximated using numerical integration but we will instead employ a Monte Carlo (MC) approach analogous to that applied elsewhere.^28^ In our case, we will generate data for each of our 432 scenarios as if they were from a huge, perfectly run RCT with full compliance and no censoring. The simulated survival times from these RCTs are then used to obtain all the causal contrasts we require, on both the hazard and survival scales, for each scenario. Specifically, for each scenario:

1. we draw 2,000,000 covariates $C_{M}$ and $C_{U}$ from the data generation model;
2. we then randomly allocate subjects to treatment X=1 or no treatment X=0 independently of these covariate values;
3. survival times, T , are then generated for each subject based on their (randomised) treatment assignment and covariate values; and
4. unadjusted Cox and unadjusted additive hazards regression models are fitted to T given X , to obtain the MC approximations of the true marginal causal hazard ratio and hazard difference. Conditional estimands can be obtained by adjusting for the $C_{M}$ parameter in the respective models. An additional step is required to derive the ETT (see Supplemental Appendixes for additional details). Kaplan–Meier estimates provide the ‘true’ marginal survival probabilities. These MC approximations will be referred to as the true causal contrasts and survival probabilities hereafter.

Since there is no guarantee that the true hazard ratio or hazard difference is constant over time in our scenarios, Step 4 is performed for each year of follow-up ( 1,2,3,4 and 5 ) whereby patients are censored at the end of the respective year if the event of interest has not been observed.

### 2.4. Simulation study

The analysis methods were compared on simulated datasets of size 2000, 10,000 and 100,000 for each of our 432 scenarios. There were 1000 replicated datasets for each scenario except for the case of the largest sample size (100,000) where 200 datasets were considered. Our primary focus is on the marginal causal contrast, the natural target of an RCT, but we also consider conditional or subgroup effects when relevant. IV estimators are known to be more variable than conventional estimators so we are particularly interested in the bias. The following outcome measures were recorded for each analysis model $j=(A_{N},C_{N},A_{2S},A_{\mathrm{RI}},C_{2S},C_{\mathrm{St}})$ , scenario k and target parameter l :

1. Estimated treatment effect: ${\hat{\theta }}_{X_{j,k}}$
2. Standard deviation of the estimates across the replicated datasets (MC standard error)
3. MC bias: $\mathrm{Bias}({\hat{\theta }}_{X_{j,k}}{)}_{l}={\hat{\theta }}_{X_{j,k}}-\theta_{X_{k,l}}$
4. Mean squared error (MSE): $\mathrm{var}({\hat{\theta }}_{X_{j,k}}$ ) + ${\mathrm{Bias}}^{2}$
5. Relative bias: $\mathrm{Bias}({\hat{\theta }}_{X_{j,k}}{)}_{l}/\theta_{X_{k,l}}$
6. Coverage: Percentage of default 95% confidence intervals that cover the true parameter $\theta_{X_{k,l}}$
7. Power: Percentage of 95% confidence intervals that do not cover the null ( 0 ) is also considered when appropriate

where $\theta_{X}$ (omitting subscripts) is the true causal contrast evaluated as described above. So, for the multiplicative scenario $\theta_{X}$ represents the hazard ratio of treatment whilst in the additive scenario $\theta_{X}$ is the hazard difference of treatment. The estimates obtained from each analysis method are compared to their relevant causal contrast $\theta_{X}$ for each scenario and year of follow-up. In addition, the methods were also compared in terms of their marginal survival probabilities. Estimated survival probabilities within each treatment arm, exposed ( X=1 ) and unexposed ( X=0 ), are obtained from each fitted model and compared to the true survival probabilities for each scenario at 1,2,3,4 and 5 years follow-up. Average differences across the simulated datasets between predicted survival and true survival are reported along with the standard deviation and minimum and maximum observed differences.

### 2.5. Computing

Data simulation and analyses were conducted using statistical software R and Stata. Simulated survival times were obtained either by inversion using R or by numerical integration and root finding methods implemented in the survsim package in Stata.^30^ Standard methods implemented in R and Stata were used to fit the naïve additive hazards regression model^27^ and the naïve Cox model^34^ and produce survival predictions. The IV methods were all fitted using the R package ivtools (version 2.3.0 ). These methods are designed to estimate causal hazard contrasts and do not consider the estimation of survival probabilities. Baseline hazards are estimated internally in the additive models and were extracted using the predict function. For the Cox IV models, we used basehaz in R to estimate the baseline hazard and then obtained marginal estimates across all patient predictions.

## 3. Results

The results are split into two sections: the results for the treatment effect estimates, and the results for the survival probability predictions. We will focus on the results for the medium sample size ( N=10,000 ) and the largest treatment effect (Table 3) but will refer to the other sample and effect sizes in our discussions.

### 3.1. Treatment effect estimates

**Figure 1.**
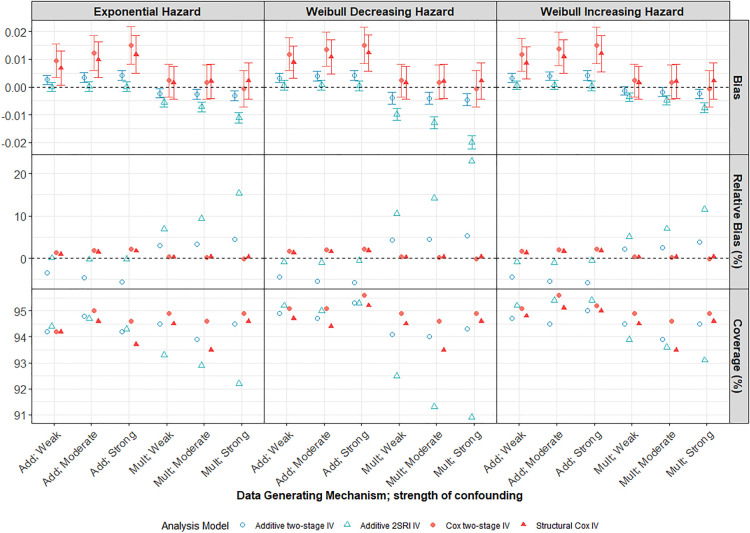
Performance of IV methods across different confounding strengths in a scenario with a large treatment effect, moderate IV and low survival probability S(5)=0.3 ( N=10,000 ). Model estimates are compared to the true marginal effect at 5 years of follow-up. Weak, moderate and strong confounding strengths are plotted across the x -axis for both Add and Mult DGMs. Each vertical panel presents a different baseline hazard distribution specified in the data-generating model. Points represent the average across the 1000 simulations. Error bars in row 1 are 95 % intervals using Monte-Carlo standard errors. Cox models are compared to the marginal hazard ratio whilst additive models are compared to the marginal hazard difference. IV: instrumental variable; DGM: data-generating mechanism; Add: additive; Mult: multiplicative.

We begin with the estimation of the marginal population causal effect, which is the target of an RCT, after 5 years of follow-up for low 5 -year survival probability ( S(5)=0.3 ). We note that a marginal effect is not the intended target of the Cox approach. Figure 1 shows results for the IV analysis models alone. Supplemental Figure D1, in Supplemental Appendix D, shows results for all six analysis models, including the naïve models. Note that the bias values on the first row refer to differences in hazard-differences (from the truth) for the additive methods, depicted by the blue symbols, and to differences of hazard ratios (from the truth) for the multiplicative methods, depicted by the red symbols. Although we show them together, they are not directly comparable which is why we also consider the relative bias.

#### 3.1.1. Impact of confounding

Both Cox and additive naïve methods (Supplemental Figure D1) were, not unexpectedly, very biased in the presence of moderate or strong unmeasured confounding and much more biased than any of the IV methods. The bias increases with confounding strength up to a relative bias of over 90 % for the additive naïve model with strong confounding under a multiplicative DGM and is reflected by the correspondingly reduced coverage probabilities. There is also very little variability in the naïve model effect estimates and so we get typically precise, but biased, estimates of the treatment effect. The outcome measures for the scenario with strong confounding and a moderate IV can be seen in Supplemental Table D1 in Supplemental Appendix D. Despite the increased variability of effect estimates, the Cox IV models had lower MSE than the naïve Cox model, regardless of the underlying DGM or baseline hazard distribution, reflecting the much greater bias in the latter. The results are less clear-cut for the additive models. Under an additive DGM, the additive IV models had lower MSE than the naïve additive model, regardless of the baseline hazard distribution despite the much lower coverage indicating the greater bias of the naïve model. However, under a multiplicative DGM, the performance depends on the baseline hazard distribution. In general, the IV estimators are more variable than the naïve estimators (Figure 1) but exhibit very little bias for all strengths of confounding provided the, respectively, assumed DGM holds.

#### 3.1.2. Mis-specification of DGM

The 2SRI additive IV method clearly outperforms the two-stage model under an additive DGM, even with strong confounding, presumably reflecting the fact that the two-stage model assumptions are not satisfied for a binary exposure. However, it is more sensitive to misspecification of the DGM and estimates are more biased than those from the two-stage additive IV model when the DGM is multiplicative. Both the two-stage and structural Cox IV models exhibited low levels of MC bias for the marginal hazard ratio under a multiplicative DGM. Importantly, the Cox IV models also seem to be less affected by misspecification of the DGM than the additive models. Notably, the structural Cox IV method which targets the ETT, appeared to be only slightly biased for the marginal hazard ratio (with a relative bias of <2.5 % even under strong confounding) and was often less biased than the two-stage Cox IV method under an additive DGM.

#### 3.1.3. Variability in IV models

**Figure 2.**
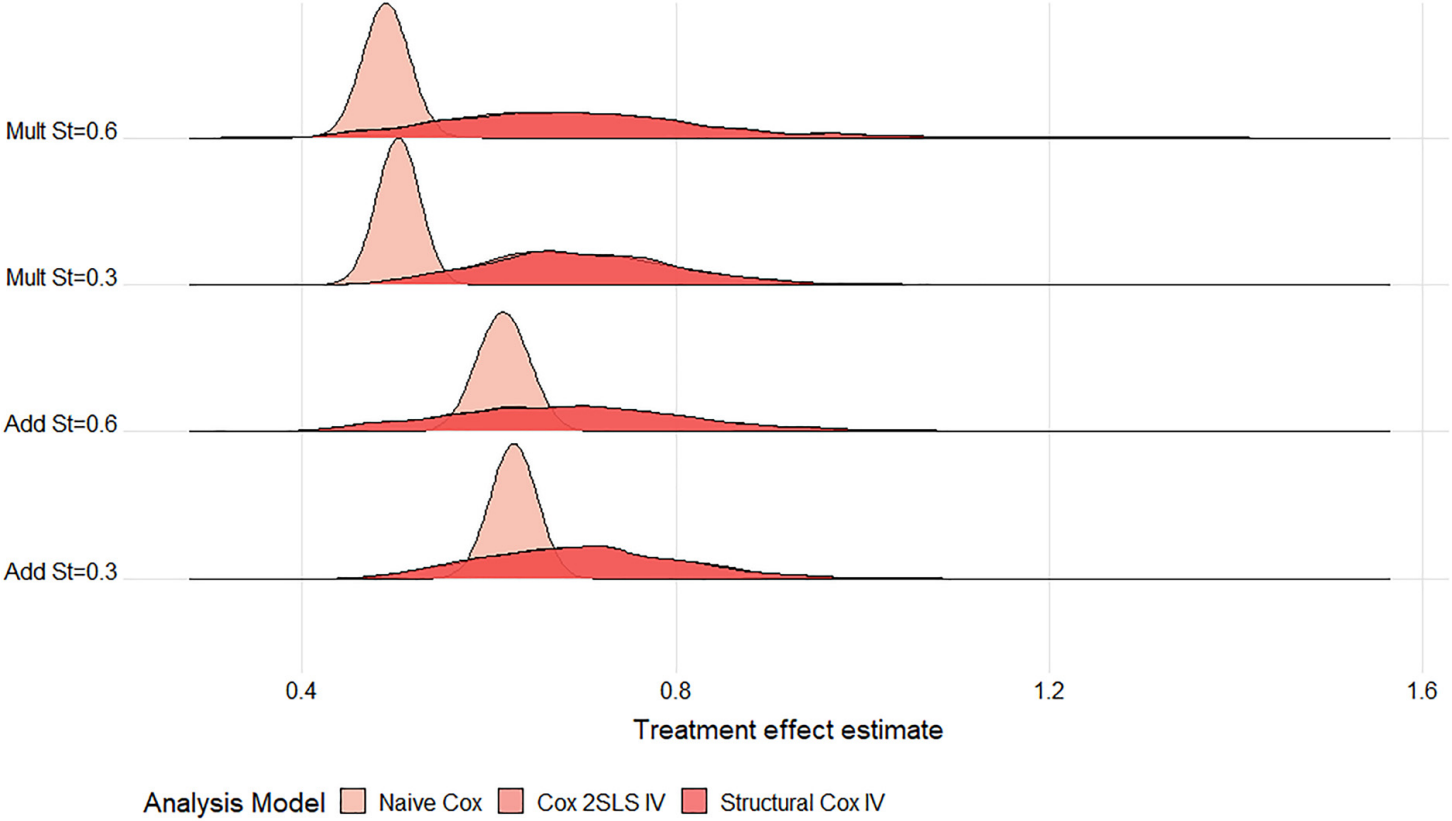
Ridgeline plot of treatment effect estimates from the Cox models across 1000 datasets for a scenario with moderate IV, strong confounding and a large treatment effect under an exponential baseline hazard at 5 years follow-up ( N=10,000 ). The DGM is given on the y -axis: Add or Mult DGM and high ( 0.6 ) or low ( 0.3 ) 5-year survival probability. The true causal HRs are: Mult S(5)=0.6 ; HR = 0.688; Mult S(5)=0.3 ; HR = 0.699; Add S(5)=0.6 ; HR = 0.683; Add S(5)=0.3 ; HR = 0.691. Note, all three curves are plotted, however, the distributions for the two IV models mostly overlap. This plot is analogous to those produced by the INTEREST tool.^35^ IV: instrumental variable; DGM: data-generating mechanism; Add: additive; Mult: multiplicate; HR: hazard ratio.

**Figure 3.**
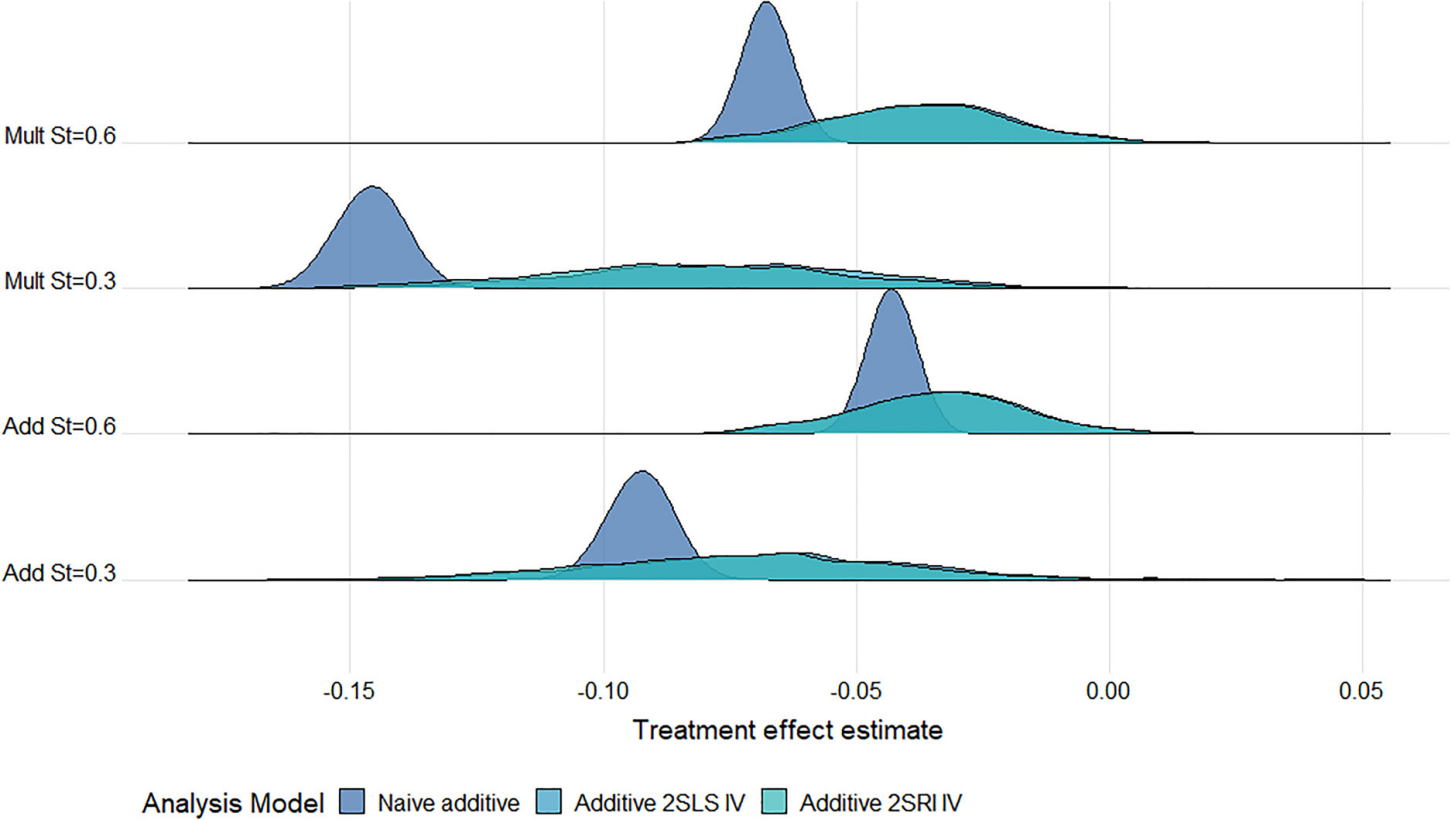
Ridgeline plot of treatment effect estimates from the additive models across 1000 datasets for a scenario with moderate IV, strong confounding and a large treatment effect under an exponential baseline hazard at 5 years follow-up ( N=10,000 ). The DGM is given on the y -axis: Add or Mult DGM and high ( 0.6 ) or low ( 0.3 ) 5-year survival probability. The true causal HDs are: Mult S(5)=0.6 ; HD =−0.034 ; Mult S(5)=0.3 ; HD =−0.072 ; Add S(5)=0.6 HD =−0.034 ; Add S(5)=0.3 HD =−0.073 . Note, all three curves are plotted, however, the distributions for the two IV models mostly overlap. IV: instrumental variable; DGM: data-generating mechanism; Add: additive; Mult: multiplicate; HD: hazard difference.

All IV estimates were variable with large MC standard errors observed for all strengths of IV and confounding. Thus, for a large proportion of the simulated datasets, the estimated treatment effect can be quite different from the truth. The difference in variability between the nai $\ddot{v}$ e and IV models is evidenced by the wide error bars in Supplemental Figure D1 (Supplemental Appendix D) and is highlighted further in the ridgeline plots of the estimates from each of the 1000 simulations (Figures 2 and 3). In general, the Cox IV methods (Figure 2) are more variable for higher 5 -year survival ( S(5)=0.6 ), perhaps due to fewer events being observed in this setting. However, this does not seem to be the case for the additive methods where estimates appear to be more variable for the lower survival setting (Figure 3). Increasing the sample size reduces this discrepancy. However, even for our largest sample size ( N=100,000 ), the additive IV estimates are much more variable for lower survival, especially when confounding is strong, and the naïve methods were both more biased for their respective causal contrasts under a multiplicative DGM (Supplemental Figures D2 to D4 in Supplemental Appendix D).

#### 3.1.4. Strength of IV

**Figure 4.**
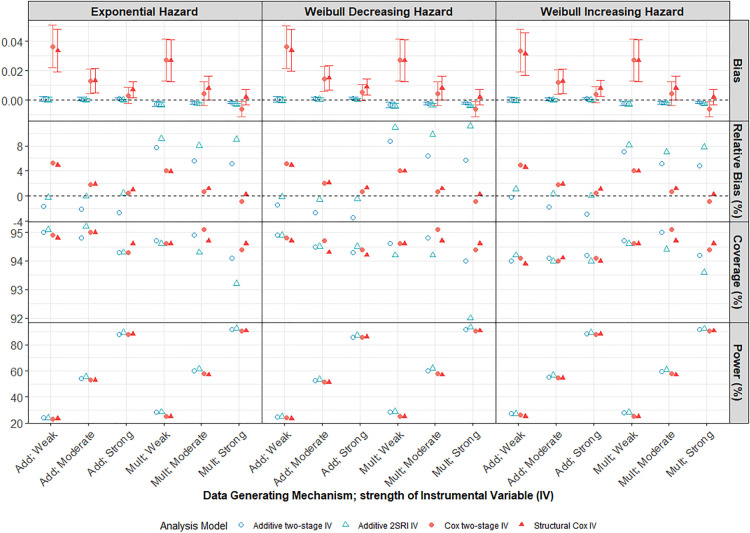
Performance of IV methods across different IV strengths compared to the marginal true parameter at 5 years follow-up. Scenario with large treatment effect, moderate confounding and low survival probability S(5)=0.3 and N=10,000 . Weak, moderate and strong instrument strengths are plotted across the x -axis for both Add and Mult DGMs. Points represent the average across the 1000 simulations. Error bars are 95% intervals using Monte-Carlo standard errors. Results for just the IV methods are presented here. IV: instrumental variable; DGM: data-generating mechanism; Add: additive; Mult: multiplicate.

Under both multiplicative and additive DGMs, the bias and variability (MC standard error (SE)) of IV methods reduce as the strength of IV increases from weak to strong (Figure 4). When the IV is very weak ( $\alpha_{Z}=0.1$ ) but importantly, still valid, all IV methods perform poorly regardless of the underlying DGM and 5 -year survival probability (Supplemental Figures D5 and D6 in Supplemental Appendix D). The structural Cox model, in particular, was noticeably more affected by a very weak IV. In fact, even for weak confounding when an IV approach may arguably not be required, IV methods can be more biased than naïve methods when the IV is very weak (Supplemental Figure D7 in Supplemental Appendix D). In such a situation, using a weak IV can actually do more harm than good in terms of circumventing suspected unmeasured confounding.^36–39^ Under an additive DGM, the 2SRI additive IV method is essentially unbiased for the marginal hazard difference, provided $\alpha_{Z}\geq 0.3$ (i.e. not very weak), but under a multiplicative DGM both additive IV methods over-estimate the hazard difference for all strengths of IV.

#### 3.1.5. Sample size

**Figure 5.**
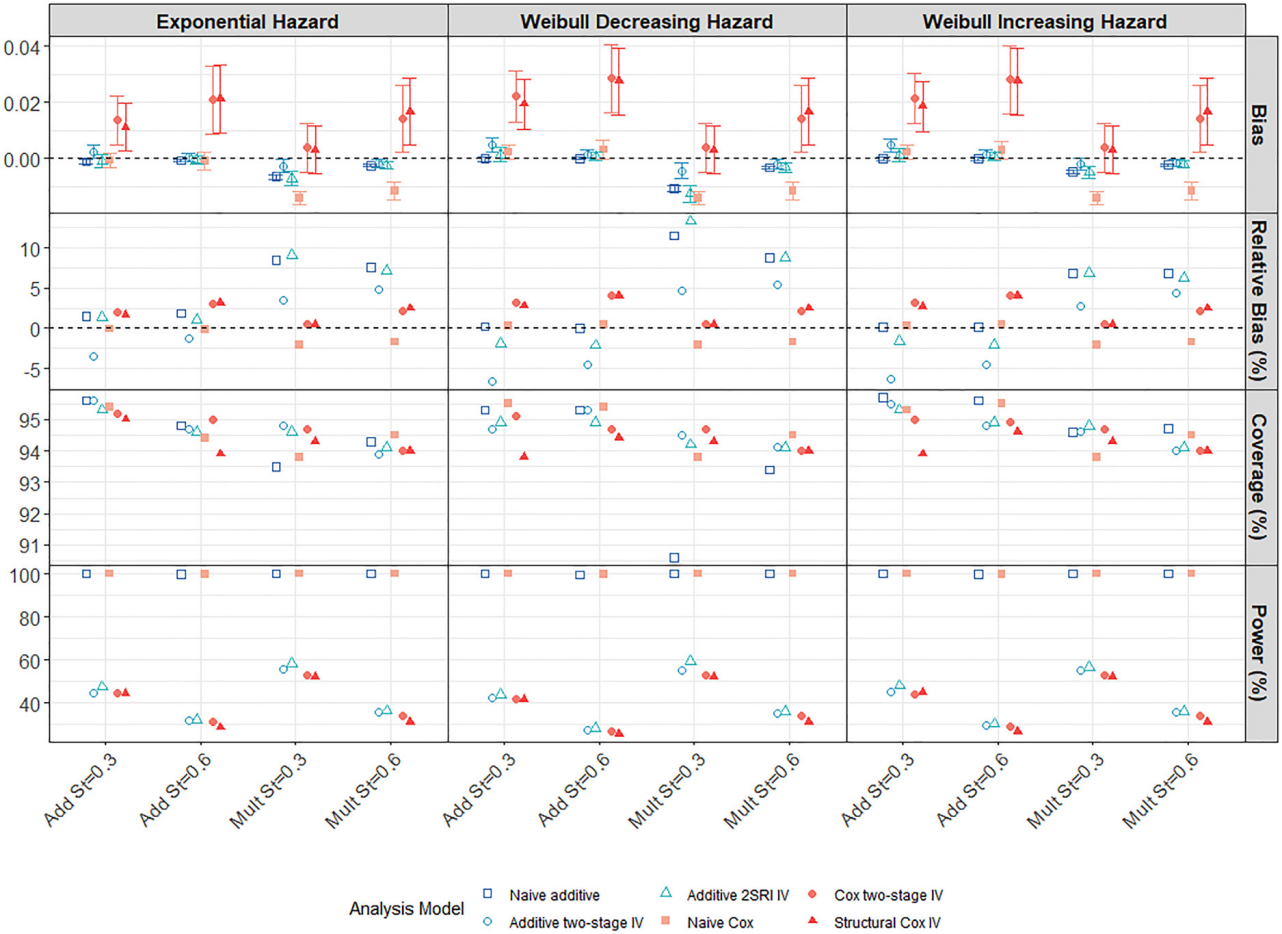
Performance of all methods compared to the true marginal effect at 5 years follow-up in a scenario with large treatment effect, weak confounding and *strong* IV ( N=2000 ). Low and high survival probabilities are plotted across the x -axis for both Add and Mult DGMs. Each vertical panel presents a different baseline hazard distribution specified in the data-generating model. Points represent the average across the 1000 simulations. Error bars are 95% intervals using Monte-Carlo standard errors. IV: instrumental variable; DGM: data-generating mechanism; Add: additive; Mult: multiplicate.

Small sample bias was evident for our smallest sample size ( N=2000 ) where IV estimators could be as biased as the naïve estimators even with a *strong* IV, weak confounding and a large treatment effect (Figure 5).

#### 3.1.6. Target causal parameter

Up to now, the discussion has focused on the marginal causal contrast which is the natural target of an RCT. However, the structural Cox IV method targets the ETT and the two-stage Cox IV model targets a conditional effect so we also considered how well these other causal contrasts were estimated. Note that the marginal and conditional/ETT causal hazard differences will not necessarily be identical under a multiplicative DGM.

The Cox methods appear to be generally more biased for their target estimands than for the marginal effect. However, as noted above, our simulation does not guarantee that there is no effect modification by Z in the multiplicative DGM. The structural Cox IV model was especially biased for the ETT under an additive DGM (see Supplemental Figures D8 and D9 in Supplemental Appendix D). Again, the 2SRI IV model was more sensitive to departures from the additivity of covariate effects than the two-stage additive IV model.

#### 3.1.7. Effect estimates over time

Finally, we also considered estimates obtained after each year 1,…,5 of follow-up. Here, the hazard ratio estimate for each year ‘ t ’ represents the hazard ratio (HR) for the interval from time 0 to the end of year ‘ t ’. Under an additive DGM and across a range of confounding and IV strengths, the 2SRI additive IV method generally out-performs the two-stage additive IV method for all years of follow-up. The Cox IV methods over-estimate the marginal causal hazard ratio for all years of follow-up under exponential and increasing Weibull baseline hazards. For a decreasing Weibull baseline hazard, they under-estimate the hazard ratio for early years and over-estimate it for later years of follow-up. Under a multiplicative DGM, the additive IV methods exhibit a large bias for early years of follow-up under a Weibull baseline hazard but this reduces as the year of follow-up increases. Variability in the IV estimates still prevails at all time points (see Supplemental Figures D10 and D11 in Supplemental Appendix D for a setting with strong confounding, large treatment effect and moderate IV).

### 3.2. Survival probability predictions

#### 3.2.1. Additive DGM

**Figure 6.**
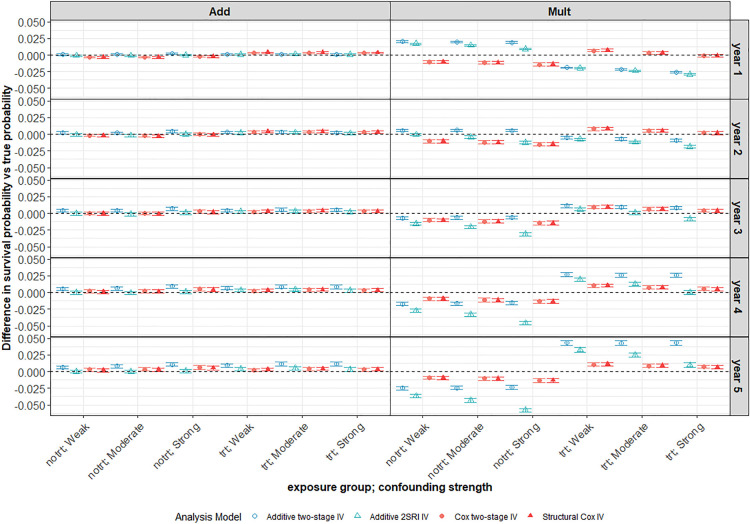
Average difference between survival probability predictions and the true marginal survival probabilities for different confounding strengths. Scenario with large treatment effect and weak IV under a decreasing Weibull baseline hazard with low 5-year survival probability ( N=10,000 ). Weak, moderate and strong confounding strengths are plotted across the x -axis for both exposure groups: trt ( X=1 ), notrt ( X=0 ). The left-hand panel of the plot depicts an additive DGM and the right-hand panel is for a multiplicative DGM. The survival probabilities were compared at years 1 to 5 of follow-up with the years presented down the right-hand y -axis. Points represent the average across the 1000 simulations. Error bars are 95% intervals using Monte-Carlo standard errors. IV: instrumental variable; DGM: data-generating mechanism; trt: treated; notrt: untreated.

Under an additive DGM, the survival probability predictions for the 2SRI additive IV and Cox IV methods exhibit very little bias across all strengths of confounding. This can be seen in the left-hand panel of Figure 6 for a decreasing Weibull baseline hazard where the average difference between predicted and true probabilities is very close to zero at all years of follow-up for the untreated ( X=0 ) group and with slight over-prediction evident in the treated ( X=1 ) group. The two-stage additive IV method over-predicts survival for later follow-up times (year 3+) and the extent of this over-prediction increases with confounding strength. Similar patterns are observed for exponential and increasing Weibull baseline hazards (Supplemental Figures D12 and D13 in Supplemental Appendix D).

#### 3.2.2. Multiplicative DGM

However, when the data are generated under a multiplicative DGM for a decreasing Weibull baseline (Figure 6 right-hand panel), the survival probability predictions appear to be biased for all IV methods and more so for the additive models. The Cox IV methods slightly under-predict survival in the untreated group and slightly over-predict in the treated group with average bias increasing slightly with the year of follow-up, but never to much more than ∼0.0125 . The additive IV methods over-predict survival in the untreated group for the early years of follow-up and under-predict survival in later years of follow-up while the reverse is observed in the treated group. The average bias increases to 0.05 at 5 years follow-up for these methods. The two-stage additive IV approach tends to be less biased in the untreated arm whereas the 2SRI additive IV method performs better in the treatment arm. There is very little difference in the performance of the Cox IV methods for different baseline hazard functions under a multiplicative DGM. However, the results for the additive IV methods are notably less consistent under the different baseline hazards and are generally better for the exponential and increasing Weibull hazards than for a decreasing Weibull hazard (Supplemental Figures D12 and D13 in Supplemental Appendix D).

#### 3.2.3. Impact of confounding

**Figure 7.**
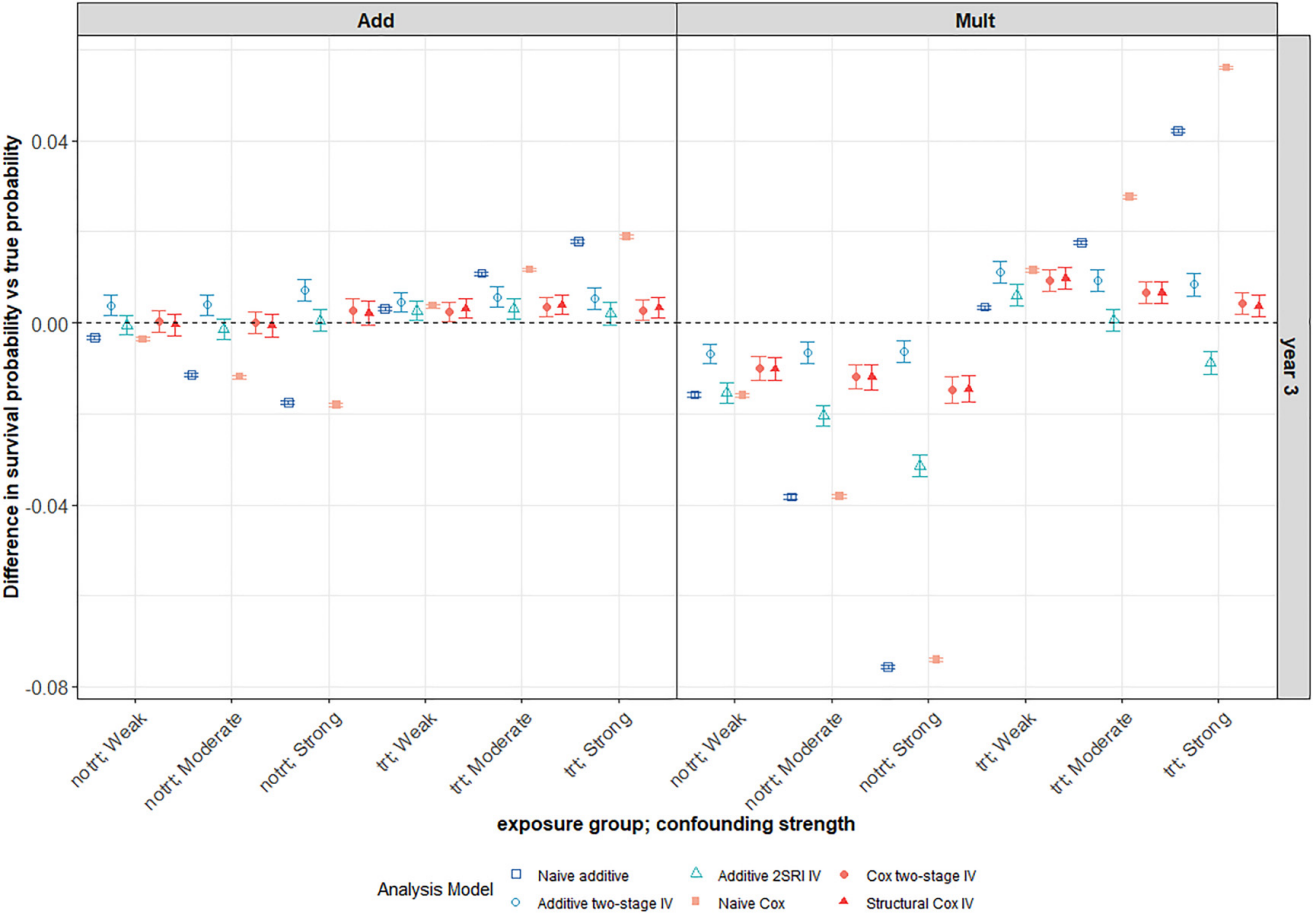
This plot focuses on the third year of follow-up for the same scenario as in Figure 6. Average differences between survival probability predictions and the true marginal survival probabilities for different confounding strengths. The survival predictions for a 3-year follow-up are plotted for both the naïve and IV methods. The scenario with large treatment effect and weak IV under a decreasing Weibull baseline hazard with S(5)=0.3 ( N=10,000 ). Weak, moderate and strong confounding strengths are plotted across the x -axis for both exposure groups: trt ( X=1 ) and notrt ( X=0 ). The left-hand panel of the plot depicts an additive DGM and the right-hand panel is for a multiplicative DGM. Points represent the average across the 1000 simulations. Error bars are 95% intervals using Monte-Carlo standard errors. IV: instrumental variable; DGM: data-generating mechanism; trt: treated; notrt: untreated.

**Figure 8.**
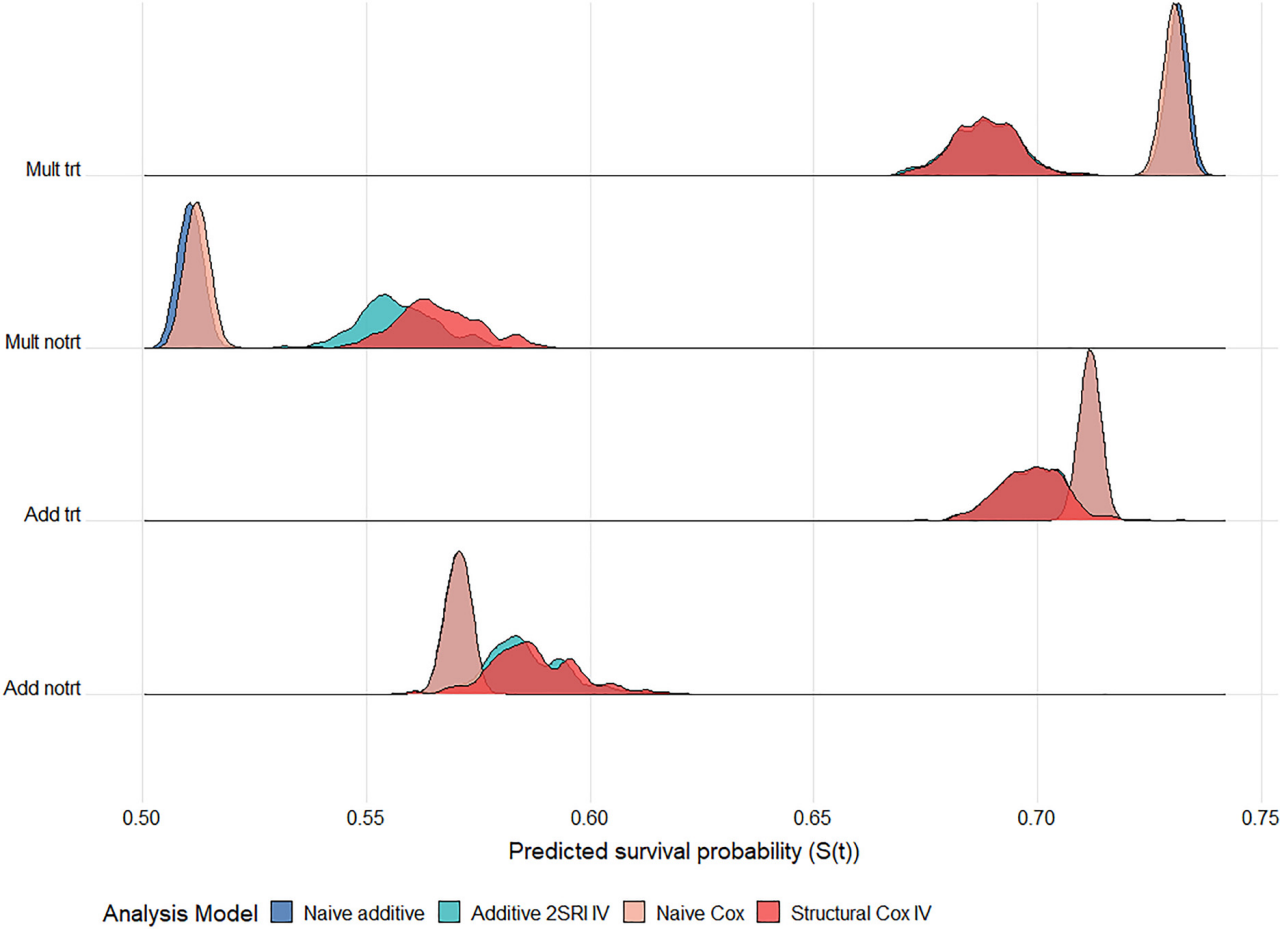
Ridgeline plot of survival probability predictions at 5 years follow up across 200 datasets for the different analysis methods. The scenario with a moderate IV, strong confounding and a large treatment effect under an exponential baseline hazard and S(5)=0.6 ( N=100,000 ). X -axis gives the survival probability predictions under each method. The DGM is given on the y -axis: Add or Mult DGM, along with the exposure arm: trt or notrt. True survival probabilities: Mult trt = 0.689, notrt = 0.582; Add trt = 0.696, notrt = 0.589. IV: instrumental variable; DGM: data-generating mechanism; Add: additive; Mult: multiplicate; trt: treated; notrt: untreated.

For all IV methods, bias in survival predictions increases slightly with confounding in the untreated arm but tends to decrease with increased confounding in the treated arm. As expected, both naïve methods give worse survival predictions in the presence of unmeasured confounding with bias increasing for stronger confounding (Figure 7). Predictions are generally worse for all methods under a multiplicative DGM. There is much more variability in survival probability predictions from all IV methods than from the corresponding naïve methods. Again, the variability problem does not go away with increased sample size, as can be seen in the ridgeline plot of Figure 8 for a scenario with 100,000 observations, although it is less extreme than for N=10,000 (Supplemental Figure D14 in Supplemental Appendix D). Generally, the estimation of survival probabilities is slightly worse in the scenarios with a higher probability of survival at 5 years ( S(5)=0.6 ) for all IV methods.

Overall, the Cox IV methods yield survival probability predictions with very little bias regardless of the underlying DGM. The 2SRI additive IV method performs best under an additive DGM but is sensitive to the DGM and to the baseline hazard function. As noted earlier, the shape of the survival curves for a Weibull hazard function and multiplicative DGM is difficult to capture using additive hazard models in our scenarios. Performance is poor for all methods and all baseline hazard functions when the IV is very weak ( $\alpha_{Z}=0.1$ ) with a notable improvement when moving to just a weak IV ( $\alpha_{Z}=0.3$ ) (Supplemental Figures D15 to D17 in Supplemental Appendix D).

## 4. Data application: Effect of statins on time-to-developing Type 2 diabetes

Statins have been found to reduce the risk of major cardiovascular events by lowering blood cholesterol.^40^ However, there are concerns that the use of statins can increase the risk of patients developing Type 2 diabetes mellitus (T2DM).^41,42^ The health problems and complications associated with T2DM may affect the benefit–risk ratio of prescribing statins to people with low risk of cardiovascular disease. A previous study found the rate of developing T2DM to be 57% (95% CI [54%, 59%]) higher in patients on statins compared to those not on statins.^43^ These results may be affected by unmeasured confounding factors. We conduct a similar analysis but using IV methods to obtain estimates of the effect of statins on the development of T2DM.^44^

### 4.1. Study design

This is an observational population-based retrospective cohort study of 650,107 patients aged 30 to 85 years between 1 January 2000 and 31 December 2018 using data from the CPRD. The primary exposure is statin use as determined using general practitioner (GP) prescriptions. Statin users were matched to non-users based on their age, gender and GP practice. There were 227,694 exposed patients and 422,413 unexposed patients of which 16,230 and 6861 , respectively, developed T2DM during follow-up. Full details of the data extraction and sample selection are provided in John.^44^

### 4.2. Instrumental variable (IV)

A potential instrument for use in this analysis is a cardiovascular disease (CVD) risk score since patients with a higher CVD risk are more likely to be prescribed statins. The QRISK score^45^ estimates cardiovascular risk with patients classified as being clinically at high risk if their 10-year cardiovascular risk is >20%. Here, CVD risk is dichotomised as high risk ( >20% ) and low risk ( ≤20% ).

**Figure 9.**
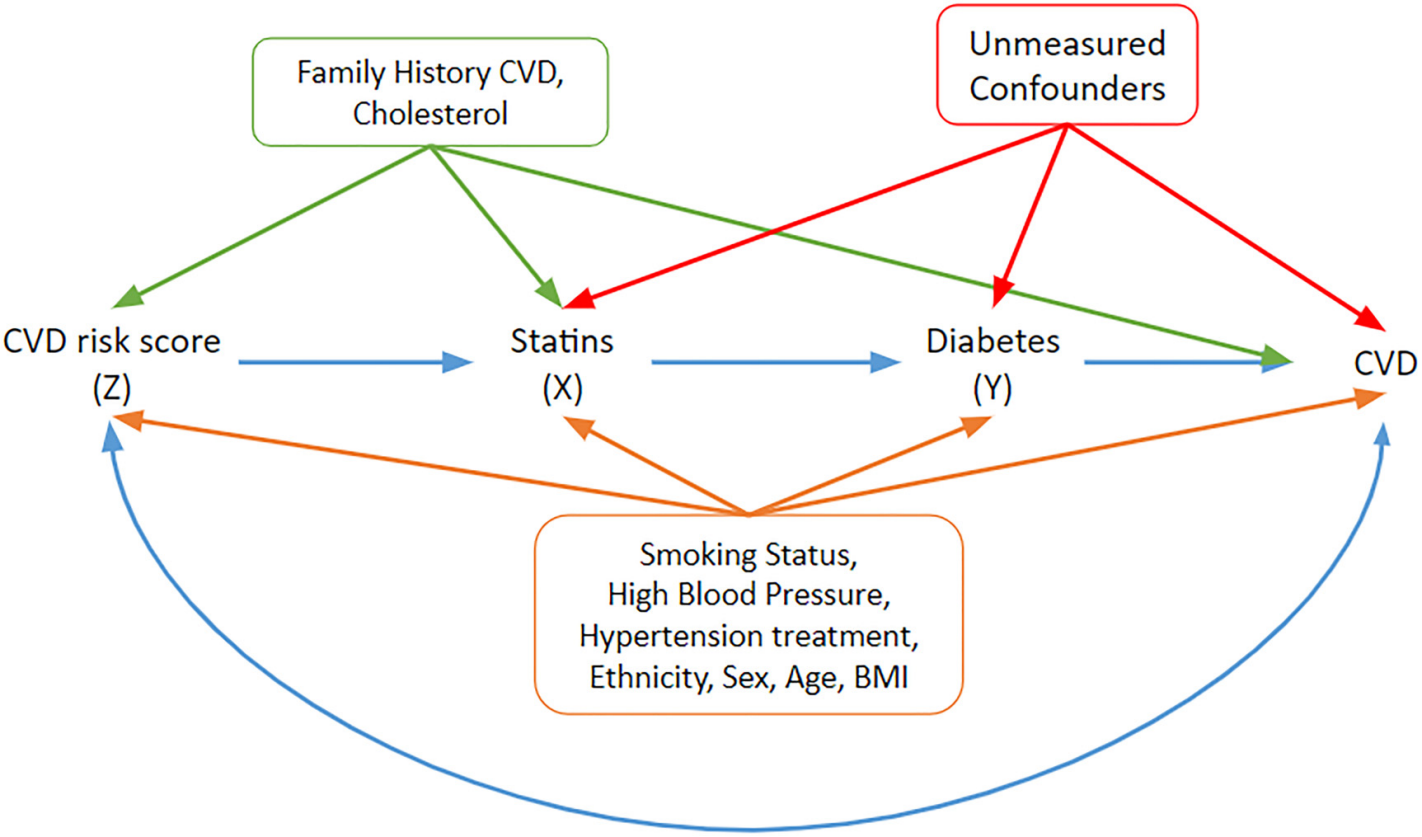
A DAG under which the graphical IV assumptions hold and under which the statin effect estimates may be interpreted as structural/causal. Relevant covariates and their potential pathways are included. The orange covariates need to be adjusted to block paths from the instrument to the outcome of interest (T2DM). The blue double-headed path represents other common causes of risk score and CVD that do not have to be accounted for. IV: instrumental variable; DAG: directed acyclic graph; CVD: cardiovascular disease; T2DM: Type 2 diabetes mellitus.

In order to assess the validity of the CVD risk score as an instrument, the predictors for the CVD score need to be compared with predictors of the outcome of interest, T2DM. The factors that are associated with T2DM are taken from the diabetes risk tool.^46,47^ Plausible associations between the different variables, the instrument Z (CVD risk score), the exposure X (statins), and outcome Y (T2DM) are outlined in a directed acyclic graph (DAG) in Figure 9.

Family history of CVD and cholesterol are associated with the instrument Z and CVD. From the current understanding of the underlying biological mechanisms,^48^ we can represent diabetes as a graph parent of CVD. Hence, CVD acts as a collider blocking all paths from Z to Y via CVD, for example, via cholesterol and family history of CVD (green arrows in the DAG) or any other common causes of risk score and CVD (blue double-headed path). All other paths between Z and Y that do not pass through X are blocked by conditioning on the variables associated with both CVD risk score and T2DM. These comprise smoking status, blood pressure, hypertension therapy, ethnicity, sex and age which are included in both CVD and diabetes risk scores, and BMI. The relevant paths are shown in orange on the DAG in Figure 9 Hence, after adjusting for the above-measured confounders, we can assume that the IV conditions are satisfied, that is, Z is associated with X (there is an unblocked path from Z to X ), Z is independent of the unmeasured confounders (no unblocked path between Z and U ) and Z is independent of Y given X and unmeasured confounders. The assumption that CVD is *not* a graph parent of diabetes is vital to justifying CVD risk score as an instrument and is based on current clinical understanding.

### 4.3. CPRD results

#### 4.3.1. Naïve analyses

Naïve additive and Cox regression models were initially fitted to the data. These models are adjusted for the main covariates: hypertension, smoking, ethnicity and BMI, which were associated with CVD risk (Figure 9) and are given in Supplemental Table E1 in Supplemental Appendix E. The naïve Cox model found there was a 4.56 95% CI ( 4.43 , 4.70 ) times higher rate of T2DM for exposed patients compared to unexposed patients.

Similar trends were observed in the naïve additive hazards model. Exposed patients had an additional hazard of 0.0099 95% CI ( 0.0097 , 0.0101 ) compared to unexposed patients. This implies that, on average, there were 9.9 additional cases of T2DM per 1000 person-years for exposed patients compared to unexposed patients.^49^

#### 4.3.2. IV analyses

In order to assess the strength of the CVD risk score as an instrument, logistic and linear regression models of exposure (statin use) on an instrument, adjusted for the measured confounders identified in Figure 9 were fitted. The logistic regression model showed that patients with high 10-year CVD risk had 3.63 95% CI ( 3.59,3.67 ) times higher odds of being prescribed statin treatment compared to patients with low 10-year CVD risk (Supplemental Table E2 in Supplemental Appendix E). The F-statistic from the linear regression model was 15,040 which would suggest that the instrument is very strong. However, the large sample size needs to be taken into account here.

Alternatively, as seen in the simulation study, the probability of exposure for the different IV groups can be compared to assess the strength of the instrumental variable. In this study, 32.7 % of patients with low 10-year CVD risk ( Z=0 ) were exposed compared to 67.1 % of patients with high 10-year CVD risk ( Z=1 ) being exposed. The difference in probability of exposure between the IV groups is 34.4 percentage points. This is a stronger IV than any used in the simulation study where the strongest IV corresponded to a difference of 28 percentage points between the two groups.

The results for the different IV models are presented in Supplemental Table E3 in Supplemental Appendix E. Both Cox IV models suggested that exposure had a causal effect on the development of T2DM but the magnitude of the effect varied depending on the model chosen. The structural Cox model found a causal effect of exposure with a 6.91 95% CI (6.67, 7.13) times higher rate of T2DM. In comparison, the effect of exposure was much smaller in the two-stage Cox model with a 2.5 95% CI (2.13, 2.99) times higher rate of T2DM.

A 2SRI additive IV model was also fitted to the data and yielded an additional hazard of 0.0129, 95% CI (0.0107, 0.0151) implying that, on average, there were 12.9 additional cases of T2DM per 1000 person-years if everyone were exposed compared to if nobody were exposed. The two-stage additive IV model estimated a slightly smaller exposure effect with an additional hazard of 0.0114, 95% CI (0.0098, 0.0130).

#### 4.3.3. Survival predictions

**Figure 10.**
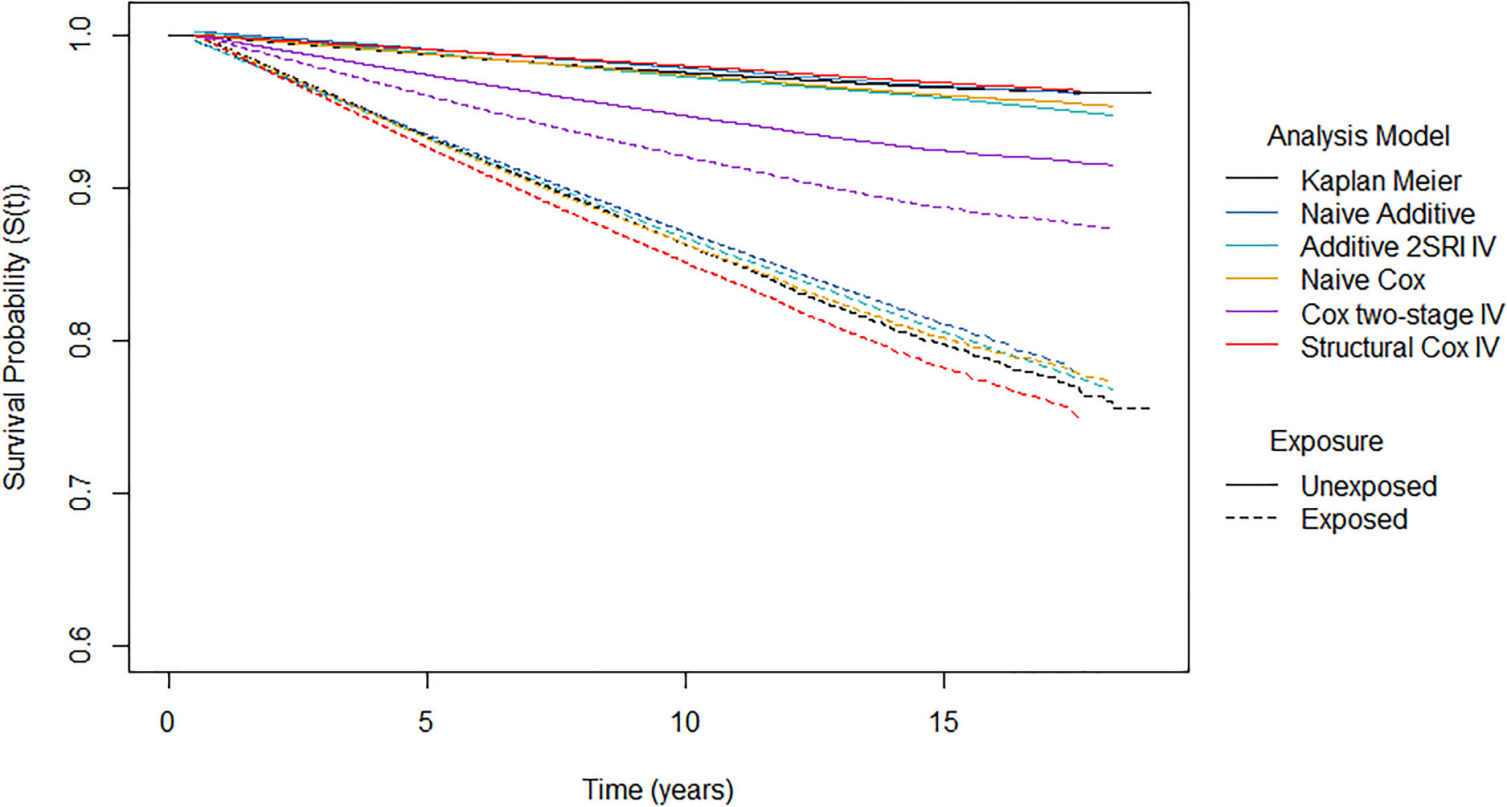
Survival probability predictions from different analysis models by exposure group. Note the y -axis had been trimmed to (0.6–1) so that the differences between model predictions are easier to see.

The survival probability predictions, which represent the probability of being diabetes free, are shown in Figure 10 for the different analysis models.

The naïve Cox, naïve additive and 2SRI additive IV models yield similar standardised survival predictions in both exposure arms. In the unexposed group, the proportion of patients that were diabetes free after 10 years of follow-up was estimated to be 97.4% for the naïve Cox model, 97.8% for the naïve additive model and 97.2% for the 2SRI additive IV model. In comparison, a lower proportion of patients were estimated to be diabetes free after 10 years of follow-up in the exposed arm: 86.3% for the naïve Cox model, 87.1% for the naïve additive model and 86.7% for the 2SRI additive IV model. This suggests that the probability of developing diabetes after 10 years is 10% higher if everyone were exposed compared to if nobody were exposed. It should be noted that we only obtained predictions at five time points from the 2SRI additive IV model due to computational issues.

The two-stage Cox IV model has much lower survival under no exposure (94.7%) and much higher survival under exposure (92.1%) compared to the other models. This reflects the much smaller hazard ratio that was obtained from the two-stage Cox IV model. The structural Cox IV model obtains similar predictions under no exposure as the other analysis models with a 10-year survival of 97.9%. However, lower survival is predicted under exposure compared to other analysis models with a 10-year survival of around 85.1%. This suggests that the probability of developing diabetes under exposure to statins is 13% higher than if the same patients had not been exposed.

All models found that statin exposure increased the risk of T2DM with none of the confidence intervals covering the relevant null effect. Not surprisingly, however, the magnitude of the effect was dependent on the analysis model used. For the most part, the IV models resulted in a larger effect of exposure on T2DM than the naïve models. Only the two-stage Cox IV model delivered a smaller exposure effect although this model is not guaranteed to target a causal estimand. The additive IV models exhibited more uncertainty about their effect estimate compared to the naïve additive regression models, as can be seen from the standard errors reported in Supplemental Table E3 in Supplemental Appendix E. This increased uncertainty was also reflected in the increased MC errors in the simulation study. The uncertainty under the structural Cox model was much more comparable to that of the naïve Cox model for this dataset whereas it was much more variable than the naïve model in the simulation study. The findings support the hypothesis of a positive causal effect of statin use on the development of T2DM but we should be wary about drawing firm conclusions about the actual size of this effect.

## 5. Discussion

RCTs remain the preferred choice for causal treatment effect estimation because randomisation ensures that confounding factors, measured or unmeasured, do not affect the estimates. However, some clinical outcomes of interest cannot be observed in an RCT and estimates from observational data are required. It is important to verify how reliable such estimates are likely to be. Standard adjustment approaches can be misleading because they fail to account for unmeasured confounders. IV methods *can* account for these and produce unbiased causal effect estimates in some situations but they rely on strong assumptions that cannot always be verified from the data. IV estimators, such as the well-known two-stage least squares (2SLS) estimator, are well developed for continuous outcomes assuming a linear additive model with no interactions but application is more problematic for non-linear models.

Our focus is on treatment effect estimation for survival, or time-to-event outcomes for a binary treatment indicator. In this study, we compared additive hazards and multiplicative hazards (Cox) IV methods, the relative merits of which, to our knowledge, have not been fully considered before. The performance of the methods was evaluated on a wide range of scenarios where the strength of confounding, strength of instrument, size of treatment effect and baseline hazard distribution were varied. Data were simulated under both additive and multiplicative DGMs so that the methods could be compared when their assumptions about the underlying DGM do and do not, hold. A truly fair comparison is difficult as these are fundamentally different models, operating under different model assumptions, different scales and targeting different causal estimands. Our approach was to generate additive and multiplicative DGMs matched on 5 -year survival probability for each scenario that we considered. One challenge was to simulate realistic survival data under an additive DGM for the different baseline hazard functions we considered as there is no restriction on the model to ensure that the hazard remains positive at all time points. Hence, different coefficient values were required for the additive and multiplicative DGMs in each case. Moreover, since the additive hazard function tended to increase much more quickly than the corresponding multiplicative hazard, we had to restrict the maximum follow-up length in the additive case to obtain larger survival probabilities at the end of follow-up and then re-scale the simulated times to get matching follow-up length. While undoubtedly far from perfect, we feel that this study goes further than previous simulation studies evaluating IV methods in time-to-event data as we compare additive and multiplicative IV methods in the same, or very similar, settings. Of course, our conclusions may not apply to scenarios where there may not be a matching multiplicative setting or where the DGM is neither additive nor multiplicative.

Despite their differences, it is natural in the context of survival analysis to expect that all the methods should predict survival, so we also compared their predicted survival probabilities with the true probabilities. Most IV analyses are designed to estimate causal hazard contrasts and do not consider survival probabilities. However, reporting survival probabilities, alongside hazard contrasts, can aid interpretation and avoid some of the problems with causal inferences based on hazard function estimates and the inherent selection bias induced by conditioning on prior survival.^21,28,50–59^ We note that these predictions are not guaranteed to be causal probability estimates since the Breslow estimator, required to obtain predictions from the Cox IV methods, for example, only accounts for the measured confounders.

Under an additive DGM, the additive 2SRI, or control function, IV estimator performed well, even when survival was low, but was extremely sensitive to misspecification of additivity. Performance was particularly poor for a multiplicative DGM with a Weibull baseline hazard function where the shape of the survival curves is difficult to capture using additive hazard models. The two-stage additive IV model estimator was always biased, presumably reflecting the fact that the two-stage model assumptions are not compatible with a binary exposure. The Cox IV methods, on the other hand, were not quite as sensitive to departures from their assumption of multiplicative covariate effects with the structural Cox method generally out-performing the two-stage Cox IV method – provided the IV was sufficiently strong. The structural Cox IV method also targets a causal parameter, the effect of ETT. The two-stage Cox method does not target a causal parameter, but its performance was not much worse than that of the structural Cox method in many cases, and it is very simple to fit. A very recent paper^60^ proposes an alternative IV estimator of the marginal causal hazard ratio, which is consistent under the assumptions of a marginal structural Cox model with an additional no-effect modification assumption in the first stage regression model. We have now tested this method on our simulated scenarios and found that it performed very similarly to the structural Cox IV method in these cases. However, all IV methods exhibited great variability across the simulations in both effect estimates and survival predictions, and this variability was still quite apparent in the largest sample size we considered ( N=100,000 ). As expected, performance improved with less confounding, larger treatment effects and stronger IVs. Note that, as in non-survival settings, an IV estimator may actually be inferior to a naïve estimator when there is little or no confounding as it will typically be more variable.

We verified that the IV should not be very weak, even with weak confounding where an IV approach is not really required. Again, performance could actually be worse than that of the corresponding naïve methods which ignore the problem of unmeasured confounding that we aim to address. There is a limit on how strong an IV can be when the unmeasured confounding is strong and thus we need to account for weak IVs in practice.^61^ We also require very large sample sizes. This is consistent with known issues for the 2SLS estimator when the IV is weak and the sample small.^36^ However, what constitutes ‘small’ depends on the distributions of outcome, exposure and IV and for our time-to-event outcomes we found that 100,000 was not sufficiently large unless the IV was strong. Dependence on the assumed DGM will not be resolved by increasing the sample size. For applications using EHR data, for example, large sample sizes will not be a problem. However, unlike our simple simulated data, they will be much more complicated with many missing values and recording errors. Methods for imputing missing data and correcting errors will be crucial to exploiting these resources and gaining the sample sizes we potentially require.

There are a few limitations and practical issues to be considered. This simulation only applied routine censoring at the end of the follow-up. Previous studies generated censoring times that follow an exponential distribution depending, in some scenarios, on the measured covariates.^19,20,25^ A more complex censoring mechanism would most likely increase bias in the effect estimators, especially if censoring were to depend on the exposure.^19^ We also generated two simple normally distributed covariates with no interactions between the covariates and treatment. Clearly, more complex covariate patterns with interactions and time-dependent effects would be more realistic. In our CPRD application, for example, it is quite likely that there would be time-dependent effects. However, our aim was to compare the performance of IV methods in settings matched on 5 -year survival probability under both additive and multiplicative DGMs. This was difficult to achieve even in the more straightforward case and would be far more challenging for more complex censoring and covariate patterns. Further investigation would be required to determine whether the patterns of behaviour observed in this study are consistent in different simulation scenarios. We should note that our simulated data did not directly favour any of the IV models. Our treatment variable was generated from a probit model although all analysis models fit a first-stage logistic regression model. Other mis-specifications should be investigated. We also did not try to ensure that the no effect-modification by Z assumption, required for the structural Cox model, was satisfied. (see Wang et al.^60^ for a more complex simulation satisfying the requirements of a marginal structural Cox model.)

Our study focused on scenarios where there is a valid IV. No IV method can be relied upon to perform well if the IV is invalid.^39,62,63^ However, finding an IV is not always easy and finding a suitably strong one is even less so. In observational situations, non-randomised instruments such as our CVD risk score have to be justified based on the available background knowledge. Background information can, of course, change over time so it should be reviewed for every new analysis. In MR applications, the genetic variants used as IVs can often be validated convincingly if there is good biological information available. Genetic IVs are typically weak. However, it is sometimes possible to combine multiple IVs into genetic risk or allele scores which can yield a stronger IV provided all the individual IVs are themselves valid.^64–66^ There is often very little choice for IVs in health services research applications where measures such as physicians’ prescribing preference, calendar time or distance to facility are typically used.^67,68^ They may or may not be strong and are difficult, if not impossible, to combine.

In several scenarios, the additive hazards IV methods gave survival predictions outside the 0 to 1 range under both additive and multiplicative DGMs (see Supplemental Figure D18 in Supplemental Appendix D for an example). This happens when the estimates of the hazard obtained for some of the event times are negative. When first proposing the model, Aalen^24^ highlighted that the hazard is not restricted to non-negative values and consequently implausible survival functions can arise.^27^ Our data were simulated to reflect biologically plausible scenarios and the naïve additive hazards model yielded valid survival predictions. Invalid predictions typically arose for the weakest IV ( $\alpha_{Z}=0.1$ ) with a frequency as high as 8% in some high survival settings, even with weak confounding. The structural Cox IV method also failed to find a solution in up to 1.3% of these scenarios. It is generally believed that this should not be an issue if very weak IVs are avoided. However, we observed invalid predictions in our CPRD application despite having a strong IV and a very large sample size. We would recommend that IV software packages output an error or warning message when negative hazard estimates arise and that they should include positivity constraints.

It is important to note that some IV programs that are in current circulation, such as those in the ivtools package, are not set up for very large datasets with multiple measured covariates. This was generally not a problem for the simple models we considered in our simulation study, although the additive models took noticeably longer to fit for the largest sample size. We were also using a high-performance computing cluster. However, there were issues with some models in the CPRD data application due to the computational size of the required predictions. Importantly, survival predictions are not automatically provided by these programs so post-estimation commands are required in order to obtain these for all the IV models we considered. In short, although there is clearly great potential for these methods in large time-to-event data applications, they will not be attractive to practitioners until some of these technical issues have been resolved. Being able to conduct such analyses on a standard desktop or laptop computer would also seem to be essential.

IV methods rely heavily on their underlying assumptions which need to be carefully considered and justified.^4,33^ In summary, of the methods we have examined, we would suggest the 2SRI estimator when an additive DGM is considered. Otherwise, the structural Cox method should be used as it also targets a causal parameter, even though the two-stage Cox is easier to implement. However, even when exhibiting very little bias, IV estimators can be highly variable and this uncertainty is often reflected in wide confidence intervals spanning the null effect. It seems that the correct DGM is just as important as having a strong IV, a large sample and a sufficient number of events (low survival). Choosing between a multiplicative hazards regression model and an additive hazards regression model requires knowledge of the effect of the exposure (multiplicative or additive) on the hazard of death. Since this is typically unknown, it makes sense to conduct sensitivity analyses by considering both classes of model and reporting survival probabilities. Even for IV analyses, standard checks and diagnostic tools for optimal model fitting, such as those recommended for naïve multiplicative and additive hazards regression models^69^ should be used, bearing in mind that the structural model itself cannot be verified from observational data.

## Supplemental Material

sj-pdf-1-smm-10.1177_09622802241293765 - Supplemental material for Multiplicative versus additive modelling of causal effects using instrumental variables for survival outcomes – a comparison Supplemental material, sj-pdf-1-smm-10.1177_09622802241293765 for Multiplicative versus additive modelling of causal effects using instrumental variables for survival outcomes – a comparison by Eleanor R John, Michael J Crowther, Vanessa Didelez and Nuala A Sheehan in Statistical Methods in Medical Research
