# Supplementary material for: Multiplicative versus additive modelling of causal effects using instrumental variables for survival outcomes – a comparison
Source: Stat Methods Med Res. 2024 Dec 10;34(1):3–25. doi: 10.1177/09622802241293765 (PMC11800712; doi:10.1177/09622802241293765)
Supplement: sj-pdf-1-smm-10.1177_09622802241293765 - Supplemental material for Multiplicative versus additive modelling of causal effects using instrumental variables for survival outcomes – a comparison [file sj-pdf-1-smm-10.1177_09622802241293765.pdf]

Appendix

Treatment probabilities for different strengths of IV

Table 5 gives the probability of treatment under different strengths of IV for weak, moderate and strong confounding.

	Instrument Strength	Probability of treatment (Mean (SD)) Z=0	Z=1	Proportion treated (X=1)	Chi-squared value χ^2
Weak Confounding	Very Weak	49.94 (8.86)	53.98 (8.63)	52.01	18.48 (<0.001)
	Weak	49.94 (8.86)	61.60 (8.31)	56.24	166.11 (<0.001)
	Moderate	49.94 (8.86)	68.80 (7.71)	59.83	415.87 (<0.001)
	Strong	49.94 (8.86)	78.32 (6.43)	64.37	923.65 (<0.001)
Moderate Confounding	Very Weak	50.04 (13.69)	53.79 (13.39)	51.37	8.13 (0.004)
	Weak	50.04 (13.69)	61.16 (12.95)	55.41	121.57 (<0.001)
	Moderate	50.04 (13.69)	68.15 (12.10)	59.81	407.52 (<0.001)
	Strong	50.04 (13.69)	77.46 (10.26)	64.06	871.32 (<0.001)
Strong Confounding	Very Weak	50.13 (19.06)	53.52 (18.80)	51.62	6.12 (0.013)
	Weak	50.13 (19.06)	60.44 (18.29)	55.15	92.36 (<0.001)
	Moderate	50.13 (19.06)	67.05 (17.30)	58.71	287.84 (<0.001)
	Strong	50.13 (19.06)	75.99 (15.09)	63.94	802.56 (<0.001)

Table 5. Effect of changing the strength of instrument on the probability of treatment in the simulated data. Mean and SD of probability of treatment presented for each IV level. Overall proportion of treated subjects also given. Chi-squared test of IV on treatment also presented.

Matching survival curves

Calculation of parameters for additive scenario

An iterative process was used to determine the parameters required for the additive scenario to generate similar survival curves to those in the corresponding multiplicative scenarios. Subjects were randomly allocated to treatment $X_{rand} = 1$ or no treatment $X_{rand} = 0$ with X_{rand} from a binomial distribution with probability 50%. This is equivalent to what would happen in an RCT where treatment is independent of a subjects' covariate values. The steps below outline the process for the exponential baseline. The following steps were then repeated using a Weibull baseline once initial parameter values had been determined.

Step 1: Initial exploration The survival times were generated under an exponential baseline hazard. The same parameters were used for both the additive and multiplicative baseline hazard scenarios. An intercept was included in the additive scenario to ensure the hazards remained positive.

The plots for a scenario with treatment effect $\beta_X = -0.4$, weak confounding $\beta_{C_U} = 0.1$ and $\lambda = 0.139$ (corresponding to $\sim 50\%$ S(t) at 5 years in the multiplicative scenario) are given in Figure 11. An intercept parameter $\beta_{0_{add}} = 1.0$ was included in the additive scenario so that the hazards remained positive. It can be seen that these two curves are not comparable. The additive scenario has a much steeper survival curve compared to the multiplicative scenario with lower survival after 5-years. Therefore different parameters are required in the additive scenario to obtain similar survival curves as in the multiplicative scenario.

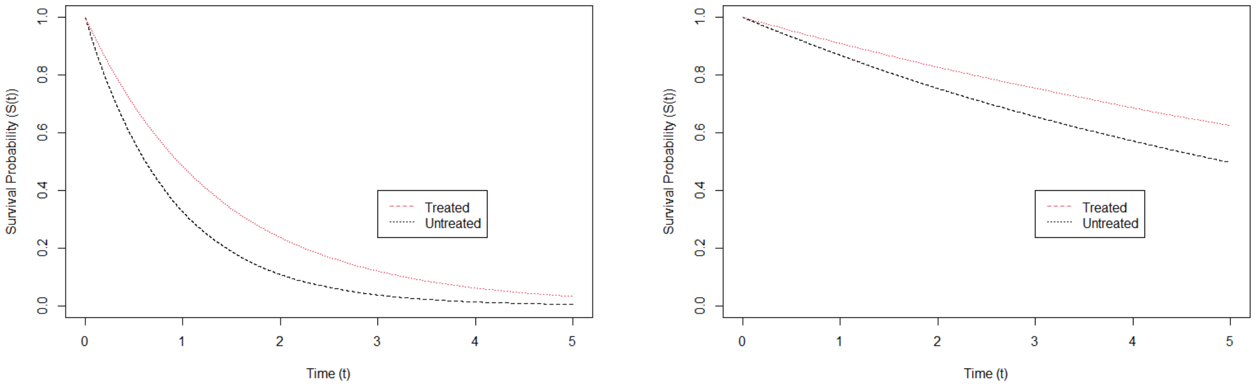

(a) Additive covariate effect **(b)** Multiplicative covariate effect

Figure 11. Survival curves obtained for a scenario with treatment effect $\beta_X = -0.4$, weak confounding $\beta_{C_U} = 0.1$ and $\lambda = 0.139$. An intercept parameter $\beta_{0_{add}} = 1.0$ was included in the additive scenario so that the hazards remained positive.

Step 2: Approximate parameters under no confounding A scenario with no confounding was used to approximate the additive treatment effect, $\beta_{X_{add}}$, and λ_{add} parameters that would give similar curves as those under a multiplicative covariate effect with parameters $\beta_{X_{mult}}$ and λ.

The parameters for the multiplicative scenario in Figure 12 were: treatment effect $\beta_{X_{mult}} = -0.4$, no confounding $\beta_{C_{U\,mult}} = 0.0$ and $\lambda = 0.139$. For the additive scenario the following parameters gave comparable survival curves as in the multiplicative scenario: treatment effect $\beta_{X_{add}} = -0.044$, no confounding $\beta_{C_{U\,add}} = 0.0$, $\lambda_{add} = -0.01$ and intercept $\beta_{0_{add}} = 0.15$.

Fitting a Cox model to the randomised data obtained an estimate of the true log hazard ratio (HR) of treatment in both scenarios. For the multiplicative scenario the true log HR was -0.3910 and for the additive scenario the true log HR was -0.3124. Therefore there is a smaller treatment effect in the additive scenario under these parameters. This can be fixed by increasing the magnitude of the treatment effect parameter $\beta_{X_{add}}$.

Step 3: Introduce covariate effects Once similar curves were found in a scenario with no confounding, covariates were introduced. The treatment effect in the multiplicative scenario was kept as $\beta_{X,mult} = -0.4$ with strong confounding introduced $\binom{\alpha_{C_{U\,mult}}}{\beta_{C_{U\,mult}}} = \binom{0.5}{0.5}$. Survival at 5 years was taken to be $S(t) \sim 60\%$ corresponding to scale parameter $\lambda = 0.102$. A small effect of the measured covariate (C_M) was also introduced with $\binom{\alpha_{C_{M\,mult}}}{\beta_{C_{M\,mult}}} = \binom{0.1}{0.1}$.

The parameters for the additive scenario were: treatment effect $\beta_{X,add} = -0.13$, confounding $\binom{\alpha_{C_{U\,add}}}{\beta_{C_{U\,add}}} = \binom{0.5}{0.05}$, $\lambda_{add} = 0.1 * \lambda$ and intercept $\beta_{0_{add}} = 0.4$. The survival curves for the above scenarios are given in Figure 13. As can be seen, the additive scenario here has much lower survival at 5 years than the multiplicative scenario. Whilst this difference is not as extreme as in Figure 11 an extra step is needed so that the survival curves are comparable.

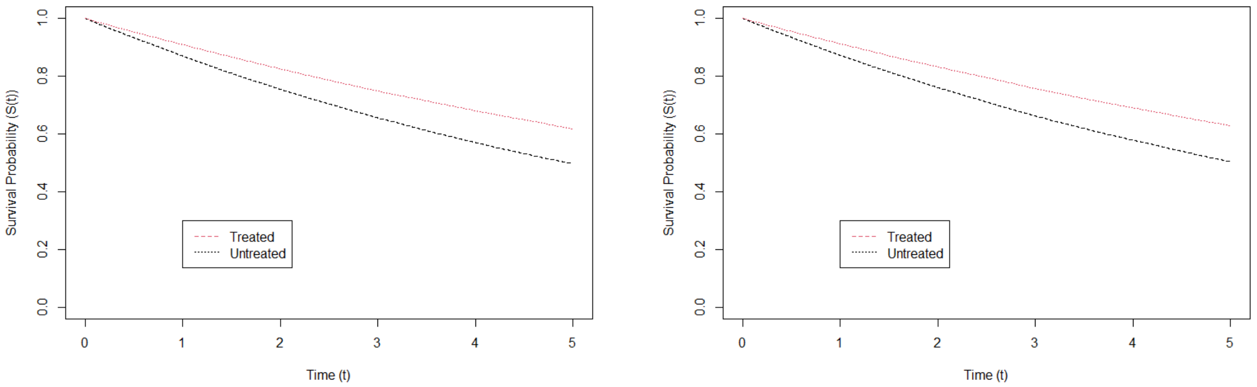

(a) Additive covariate effect

(b) Multiplicative covariate effect

Figure 12. Survival curves obtained in a scenario with no confounding. Additive parameters for (A) treatment effect $\beta_{X\,add} = -0.044$, no confounding $\beta_{C_U\,add} = 0.0$, $\lambda_{add} = -0.01$ and intercept $\beta_{0\,add} = 0.15$. Multiplicative parameters for (B) treatment effect $\beta_{X\,mult} = -0.4$, no confounding $\beta_{C_U\,mult} = 0.0$ and $\lambda = 0.139$.

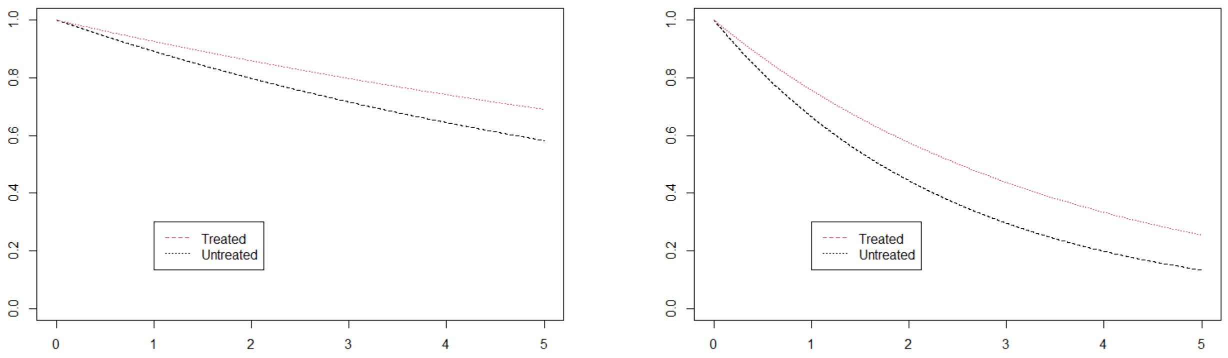

(a) Multiplicative covariate effect

(b) Additive covariate effect

Figure 13. Survival curves obtained after introducing covariates. Multiplicative parameters for (A) treatment effect $\beta_{X\,mult} = -0.4$, confounding $\beta_{C_U\,mult} = 0.5$ and $\lambda = 0.102$. Additive parameters for (B) treatment effect $\beta_{X\,add} = -0.13$, confounding $\beta_{C_U\,add} = 0.05$, $\lambda_{add} = 0.0102$ and intercept $\beta_{0\,add} = 0.4$.

Step 4: Restrict and re-scale follow-up time By restricting the follow up in the additive scenario, higher survival at the end of follow up could be obtained. To obtain a survival $\sim 60\%$ at the end of follow up, similar to the multiplicative scenario in Step 3, additive survival times were generated with a maximum follow up t of 1.3 years (Figure 14 (B)). These times can then be re-scaled by 3.846 to give the same length of follow up as in the multiplicative scenario i.e follow up of 5 years (Figure 14 (C)). Once the times have been re-scaled the survival curves are comparable to the multiplicative scenario (Figure 14 (A)).

As in Step 2, an estimate of the true log hazard ratio (HR) of treatment in both scenarios can be obtained by fitting a Cox model to the randomised data. For the multiplicative scenario the true log HR was -0.3822 and for the additive scenario the true log HR was -0.3839. Therefore the log HR are now comparable between the additive and multiplicative scenarios.

Step 5: Compare survival under observational scenario Steps 1-4 above were conducted using a randomised treatment allocation as would occur in an RCT. Here, an observational dataset using the observed treatment allocation under Equation (4) will be used. This will allow the level of confounding on the outcome to be assessed in an observational setting. Different strengths of confounding are simulated by varying the α_{C_U} and β_{C_U} parameters.

Initially the α_{C_U} and β_{C_U} parameters remained as in Step 4 above with α_{C_U} positive. As can be seen in Figure 15 (A) the confounding effect reduces the difference in survival between the treatment arms compared to the RCT scenario for the multiplicative scenario (Figure 14 (A)). Those with larger values of C_U were more likely to be treated since $\alpha_{C_U\,mult} = 0.5$. The effect of treatment in reducing the hazard $\beta_{X\,mult} = -0.4$ is then cancelled out, in those treated subjects with larger C_U, by the detrimental effect of $\beta_{C_U\,mult} = 0.5$ increasing the hazard. This is reflected in the estimated treatment effect estimate for the observed data with a log HR of $= -0.0355$ compared to the true log HR of -0.3822.

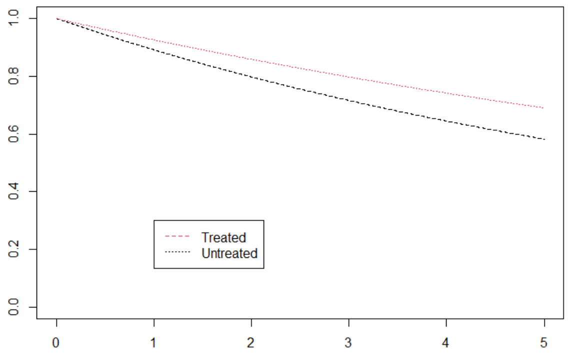

(a) Multiplicative covariate effect

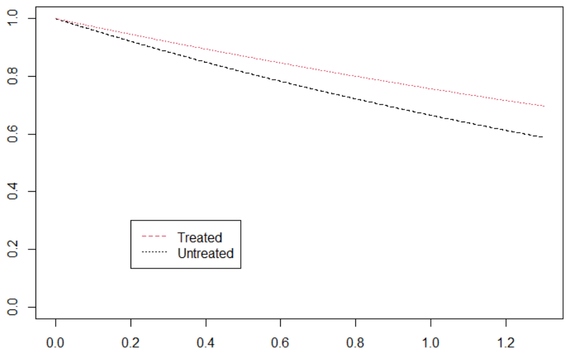

(b) Additive covariate effect: Follow-up 1.3

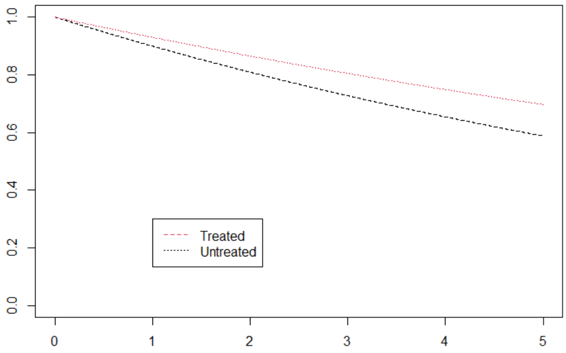

(c) Additive covariate effect: re-scaled follow-up

Figure 14. Survival curves obtained after introducing covariates and restricting follow up in the additive scenario. Multiplicative parameters for (A) treatment effect $\beta_{X\,mult} = -0.4$, confounding $\beta_{C_U\,mult} = 0.5$ and $\lambda = 0.102$. Additive parameters for (B) treatment effect $\beta_{X\,add} = -0.13$, confounding $\beta_{C_U\,add} = 0.05$, $\lambda_{add} = 0.0102$ and intercept $\beta_{0\,add} = 0.4$. Additive survival times were generated with a maximum follow up t of 1.3 years. The same additive parameters are used in (C) with the times re-scaled by 3.846 to give follow-up of 5 years.

To make the effect of confounding more apparent, the α_{C_U} parameter was made negative. In Figure 15 (B) $\alpha_{C_U\,mult} = -0.5$ which leads to a much bigger observed effect of treatment compared to the RCT scenario (Figure 14 (A)). This is reflected in the estimated treatment effect for the observed data with a log HR of -0.6885 compared to the true with a log HR of -0.3822. This scenario will be used so that confounding can be more easily detected as it will increase the observed treatment effect. When confounding cancels out the true treatment effect it is much harder to detect that the confounding is present.

A similar change occurs in the additive scenario. As can be seen in Figure 15 (C), when $\alpha_{C_U\,add} = 0.5$ the confounding effect reduces the difference in survival between the treatment arms compared to the RCT scenario (Figure 14 (C)). This is reflected in the estimated treatment effect for the observed data with a log HR of $= -0.2688$ compared to the true log HR of -0.3839. Changing $\alpha_{C_U\,add} = -0.5$ in Figure 15 (D) leads to a bigger observed effect of treatment compared to the RCT scenario (Figure 14 (C)). Again, this is reflected in the estimated treatment effect for the observed data with a log HR of -0.4786 compared to the true with a log HR of -0.3839.

Step 6: Repeat steps for different simulation scenarios The above steps yield the parameters required for a scenario with strong confounding, high survival ($S(t) \sim 60\%$) at 5 years, and an exponential baseline hazard. This simulation will look at a range of scenarios including different strengths of confounding (weak, moderate and strong), different proportions of survival at 5 years (low/high) and three baseline hazard distributions (exponential, decreasing and increasing weibull). Steps 4-5 above were repeated for each combination of the different simulation scenarios.

The covariate parameters for the multiplicative scenario that give weak, moderate and strong confounding are $\begin{pmatrix} \alpha_{C_U\,mult} \\ \beta_{C_U\,mult} \end{pmatrix} = \begin{pmatrix} -0.1 \\ 0.1 \end{pmatrix}, \begin{pmatrix} -0.3 \\ 0.3 \end{pmatrix}$ and $\begin{pmatrix} -0.5 \\ 0.5 \end{pmatrix}$. The parameters for the additive scenario that give different levels of confounding were $\begin{pmatrix} \alpha_{C_U\,add} \\ \beta_{C_U\,add} \end{pmatrix} = \begin{pmatrix} -0.1 \\ 0.01 \end{pmatrix}, \begin{pmatrix} -0.3 \\ 0.04 \end{pmatrix}$ and $\begin{pmatrix} -0.5 \\ 0.05 \end{pmatrix}$. The strength of confounding was assessed by fitting a naive model to the non-randomised data (Step 5). In general, confounding is slightly stronger in the multiplicative scenario than in the additive scenario due to the data

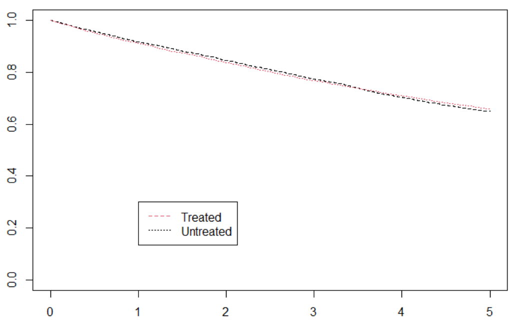

(a) Multiplicative covariate effect: positive α_{C_U}

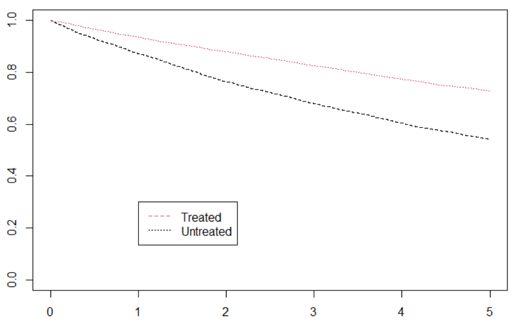

(b) Multiplicative covariate effect: negative α_{C_U}

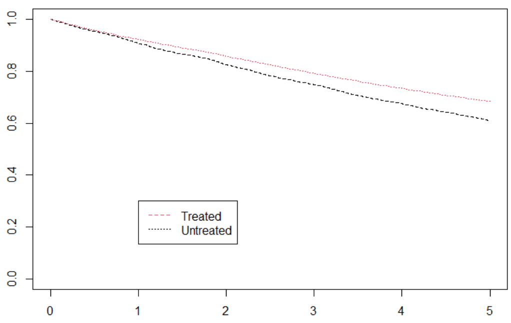

(c) Additive covariate effect: positive α_{C_U}

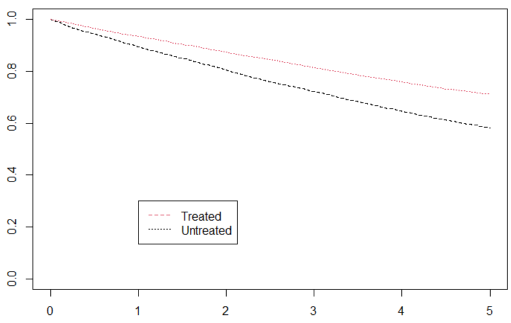

(d) Additive covariate effect: negative α_{C_U}

Figure 15. Survival curves obtained for observational treatment allocation. The confounding parameters were (A) $\binom{\alpha_{C_U\,mult}}{\beta_{C_U\,mult}} = \binom{0.5}{0.5}$ (B) $\binom{\alpha_{C_U\,mult}}{\beta_{C_U\,mult}} = \binom{-0.5}{0.5}$ (C) $\binom{\alpha_{C_U\,add}}{\beta_{C_U\,add}} = \binom{0.5}{0.05}$ (D) $\binom{\alpha_{C_U\,add}}{\beta_{C_U\,add}} = \binom{-0.5}{0.05}$.

generating mechanism. However, increasing the $\beta_{C_U\,add}$ parameter in order to increase the strength of confounding led to negative hazards being obtained. When a larger intercept was introduced, to ensure positive hazards, this changed the shape of the curves so that they were no longer comparable to the multiplicative scenarios. Therefore, it was not possible to increase confounding in the additive scenario whilst maintaining comparable survival curves as in the multiplicative scenario. The parameters above will allow for a relative increase in confounding strength for both the additive and multiplicative scenarios. Whilst confounding will be slightly weaker in the additive scenario, the survival curves will remain comparable with the multiplicative scenario.

For an exponential baseline it was possible to obtain comparable shapes of the survival curves for both multiplicative and additive covariate effects (Figure 16 (A/B)). When a Weibull baseline is used, the shape of the curves are slightly different when there is an additive or multiplicative covariate effect. This can be seen in Figure 16 where there is a steeper survival curve for the decreasing Weibull when the covariate effect is multiplicative (C) compared to when an additive covariate effect is used (D). Slight differences in the shape of the curves are also observed when an increasing Weibull baseline is used (Figure 16 (E/F)). The differences in the shape of the curves occur due to the data generating mechanism taking an additive or multiplicative covariate effect and thus it is not possible to alter the shape by changing the parameters. However, it was possible to match the survival probabilities at 5-years follow-up between the additive and multiplicative scenarios. Here the focus was on ensuring the survival probabilities at the end of follow-up were similar across the two data generating mechanisms.

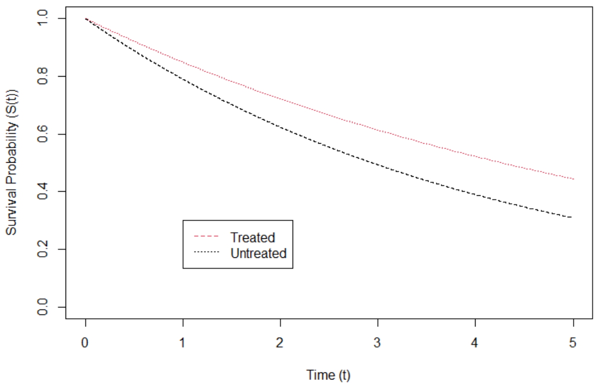

(a) Exponential baseline (multiplicative)

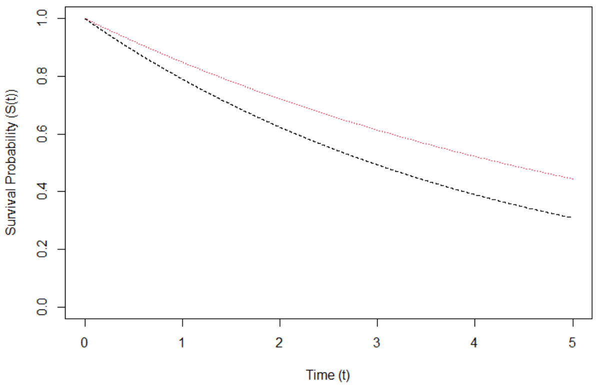

(b) Exponential baseline (additive)

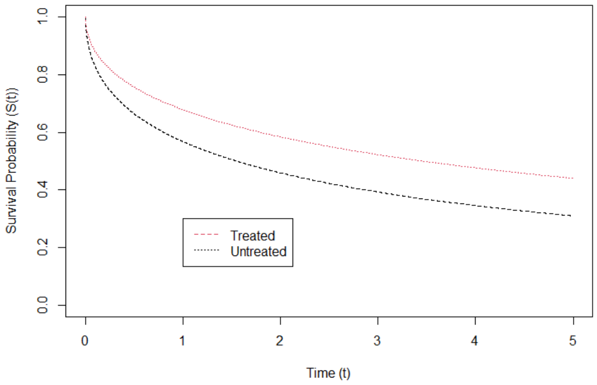

(c) Weibull Decreasing baseline (multiplicative)

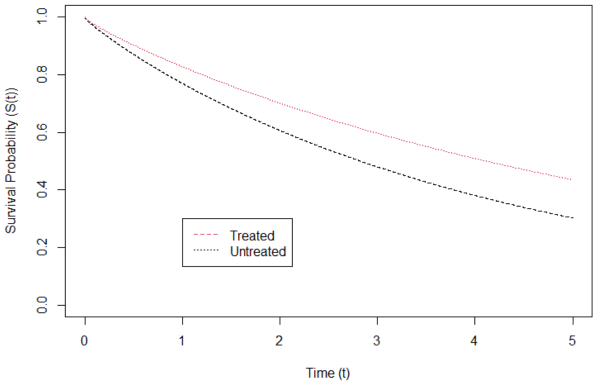

(d) Weibull Decreasing baseline (additive)

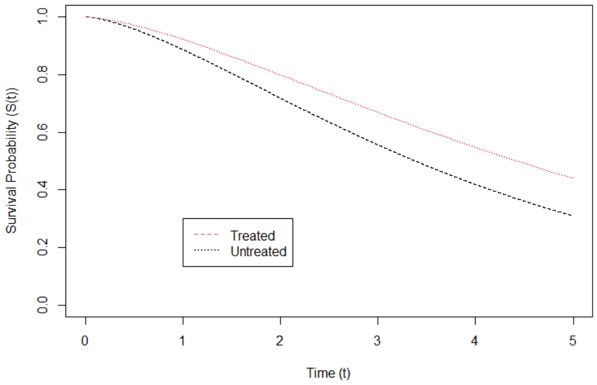

(e) Weibull Increasing baseline (multiplicative)

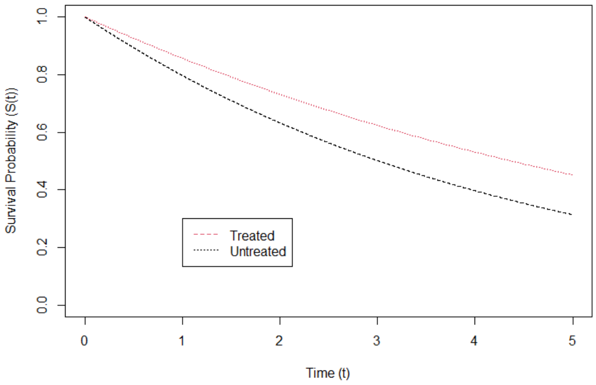

(f) Weibull Increasing baseline (additive)

Figure 16. Survival curves obtained for different baseline hazard functions: exponential, decreasing Weibull and increasing Weibull. Scenario here is strong confounding and low survival (30%).

Evaluating 'true' causal contrasts

In order to assess bias in the model-based estimators we need to obtain the true causal parameter for each scenario. The Cox and additive methods applied in this study estimate different, and thus incomparable, parameters. The Cox methods target a hazard ratio whilst the additive methods target a hazards difference. Therefore, for the Cox methods bias needs to be assessed compared to the true causal hazard ratio whereas for the additive methods bias needs to be assessed compared to the true hazard difference. Methods to calculate the true hazard ratio and true hazard difference for each simulation scenario will be discussed here.

The aim here is to simulate data as if it were from a huge perfectly run RCT with no non-compliance. The simulated times from this RCT can then be used to get the true target estimands for each scenario. The following steps are required:

1. Simulate covariates C_M and C_U based on the data generation model
2. Randomly allocate subjects to treatment $X = 1$ or no treatment $X = 0$ independent of their covariate values
3. Generate survival times T for each subject based on their randomised treatment assignment and observed covariate values
4. Fit a model to the randomised data to obtain the true causal estimands

Step 1: Simulate covariates

For a very large sample size ($N = 2,000,000$) simulate the covariates C_M and C_U from the same distributions as in the data generating model.

Step 2: Random treatment allocation

Randomly allocate subjects to treatment $X_{rand} = 1$ or no treatment $X_{rand} = 0$ so that treatment is independent of a subjects' covariate values. This is equivalent to what would happen in an RCT. To do this take X from a binomial distribution with probability 50%:

$$X_{rand} \sim Binom(0.5). \tag{5}$$

Step 3: Generate survival times

Generate survival times T for each subject based on their randomised treatment X_{rand} and covariate values C_M and C_U. It will be assumed that there is no censoring during follow-up. Subjects will be routinely censored at 5 years. Survival times can be generated using the inversion method when the survival functions are invertible. When the survival function is not invertible numerical integration and root finding methods will be used.

Step 4: Fit models

The true causal estimands can be obtained by fitting a model to the observed data as would be done in an RCT. Since the data are simulated as in a perfect RCT with no non-compliance this will give the desired true causal estimand. The marginal causal hazard ratio is obtained by fitting a Cox model including only the exposure X without any covariates. The conditional causal hazard ratio is obtained by fitting a Cox model including the exposure X and measured covariate C_M. A second conditional hazard ratio, given C_M and C_U will also be obtained. The marginal causal hazard difference is obtained by fitting an additive hazards model including the exposure X without adjusting for any covariates. The conditional causal hazard difference is obtained by fitting an additive hazards model adjusted for the exposure X and measured covariate C_M. This is not necessarily the same as the marginal hazard difference under a multiplicative DGM. A second conditional hazard difference, given C_M and C_U will also be obtained. Due to collapsibility of the hazard difference the conditional and marginal hazard differences will be the same under the additive DGM. By restricting the length of follow-up to which the above models are fit, it is possible to obtain causal hazard ratios and hazard differences at a range of follow-up times.

Step 5: Average treatment effect in the treated

The structural Cox model targets the average treatment effect within those subjects who were treated (ATT). To obtain the true ATT causal contrasts an extra step is needed.

As above, the covariates C_M and C_U are generated (Step 1). Patients' observed treatment allocation is then obtained using Equation 4. Those patients who were observed to be exposed ($X_{obs} = 1$) are then extracted. Two sets of survival times are then generated in step 3:

1. Survival times under the observed exposure where $X = 1$ for all patients
2. Survival times assuming all patients were unexposed by setting $X = 0$ for all patients

The fitted models (Step 4) will then obtain the marginal and conditional ATT causal contrasts.

Step 6: Survival probabilities

The true survival probabilities can be calculated for each simulation scenario using the Kaplan-Meier estimator of survival. This will give the marginal survival probability averaged across the covariates C_M and C_U. The true probabilities were calculated for both the exposed and unexposed treatment groups. The true probabilities were calculated at a range of follow up times $t = 1, 2, 3, 4$ and 5.

Additional simulation results

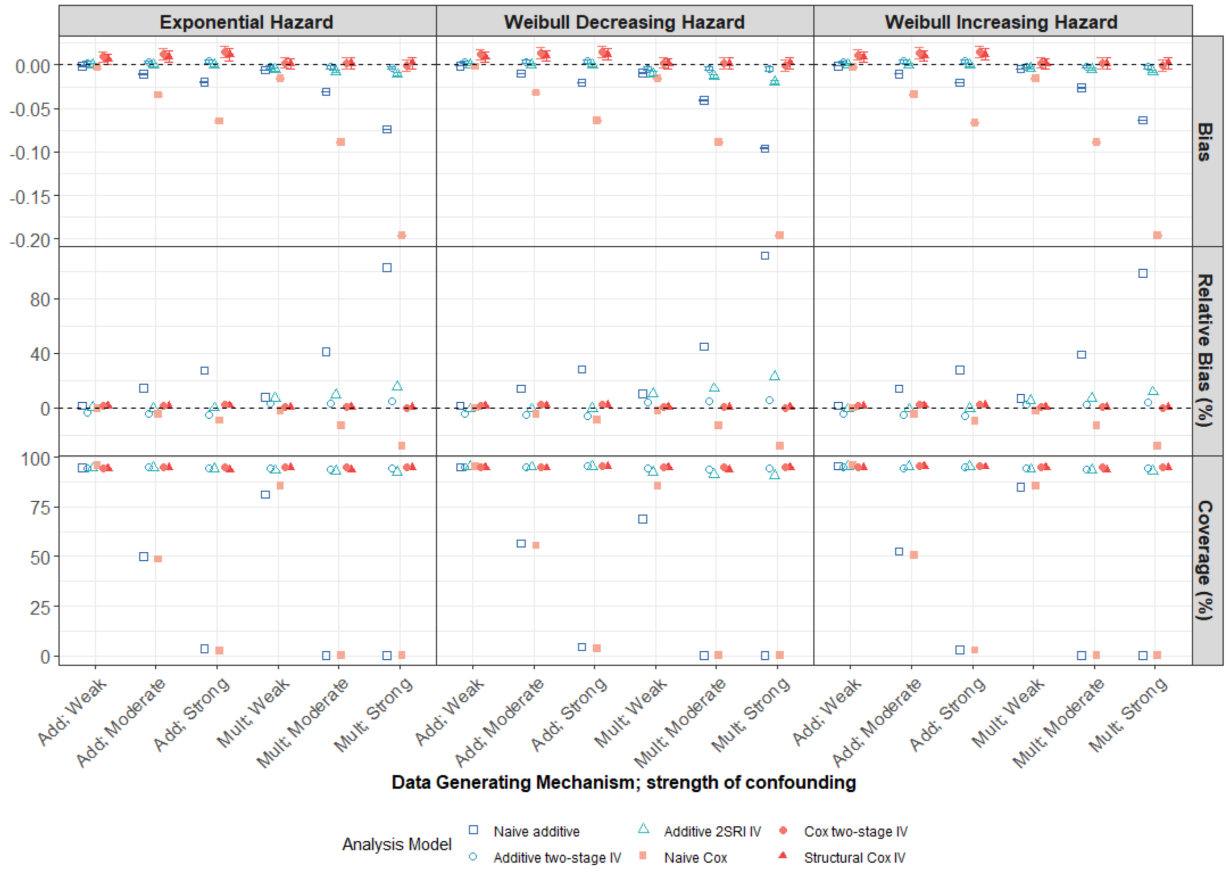

Figure 17. Performance of all methods across different confounding strengths in a scenario with a large treatment effect, moderate IV and low survival probability $S(5) = 0.3$ $(N = 10,000)$. Model estimates are compared to the true marginal effect at 5 years follow-up. Weak, moderate and strong confounding strengths are plotted across the x-axis for both additive (Add) and multiplicative (Mult) DGMs. Each vertical panel presents a different baseline hazard distribution specified in the data generating model. Points represent the average across the 1,000 simulations. Error bars are 95% intervals using Monte-Carlo standard errors. Cox models are compared to the hazard ratio whilst additive models are compared to the hazard difference.

		Exponential		Decreasing Weibull		Increasing Weibull	
Analysis Model	Parameter	Multiplicative	Additive	Multiplicative	Additive	Multiplicative	Additive
Naïve Additive	Effect Estimate	-0.14599 (0.01)	-0.09257 (0.01)	-0.18241 (0.01)	-0.09233 (0.01)	-0.12854 (0.00)	-0.09235 (0.01)
	Bias	-0.06797 (0.01)	-0.02002 (0.01)	-0.05721 (0.01)	-0.01908 (0.01)	-0.07311 (0.00)	-0.01951 (0.01)
	Mean Squared Error	0.00469 (<0.001)	0.00046 (<0.001)	0.0034 (<0.001)	0.00043 (<0.001)	0.00539 <(0.001)	0.00044 (<0.001)
	Coverage, n (%)	0 (0.00)	32 (3.20)	0 (0.00)	66 (6.60)	0 (0.00)	46 (4.60)
	Power, n (%)	1000 (100.00)	1000 (100.00)	1000 (100.00)	1000 (100.00)	1000 (100.00)	1000 (100.00)
Two-stage Additive IV	Effect Estimate	-0.07522 (0.03)	-0.06862 (0.03)	-0.09095 (0.04)	-0.06793 (0.03)	-0.06728 (0.03)	-0.06809 (0.03)
	Bias	0.0028 (0.03)	0.00393 (0.03)	0.03426 (0.04)	0.00532 (0.03)	-0.01184 (0.03)	0.00475 (0.03)
	Mean Squared Error	0.00174 (<0.001)	0.00171 (<0.001)	0.00368 (<0.001)	0.00171 (<0.001)	0.00154 (<0.001)	0.00156 (<0.001)
	Coverage, n (%)	948 (94.80)	941 (94.10)	839 (83.90)	952 (95.20)	918 (91.80)	950 (95.00)
	Power, n (%)	725 (72.50)	681 (68.10)	726 (72.60)	644 (64.40)	721 (72.10)	687 (68.70)
2SRI Additive IV	Effect Estimate	-0.08305 (0.03)	-0.07262 (0.03)	-0.10623 (0.04)	-0.07173 (0.03)	-0.07228 (0.03)	-0.07191 (0.03)
	Bias	-0.00503 (0.03)	-7e-05 (0.03)	0.01897 (0.04)	0.00152 (0.03)	-0.01685 (0.03)	0.00093 (0.03)
	Mean Squared Error	0.00193 (<0.001)	0.00176 (<0.001)	0.00327 (<0.001)	0.00173 (<0.001)	0.00178 (<0.001)	0.00158 (<0.001)
	Coverage, n (%)	942 (94.20)	943 (94.30)	921 (92.10)	952 (95.20)	886 (88.60)	954 (95.40)
	Power, n (%)	784 (78.40)	711 (71.10)	820 (82.00)	677 (67.70)	761 (76.10)	716 (71.60)
Naïve Cox	Effect Estimate	-0.68776 (0.02)	-0.46798 (0.03)	-0.68776 (0.02)	-0.44958 (0.03)	-0.68776 (0.02)	-0.47761 (0.03)
	Bias	-0.31758 (0.02)	-0.10101 (0.03)	-0.32281 (0.02)	-0.10406 (0.03)	-0.31277 (0.02)	-0.096 (0.03)
	Mean Squared Error	0.1021 (0.02)	0.01152 (0.01)	0.10546 (0.02)	0.01215 (0.01)	0.09907 (0.02)	0.01058 (0.01)
	Coverage, n (%)	0 (0.00)	22 (2.20)	0 (0.00)	23 (2.30)	0 (0.00)	44 (4.40)
	Power, n (%)	1000 (100.00)	1000 (100.00)	1000 (100.00)	1000 (100.00)	1000 (100.00)	1000 (100.00)
Structural Cox IV	Effect Estimate	-0.36557 (0.15)	-0.36426 (0.15)	-0.36556 (0.15)	-0.34649 (0.15)	-0.36557 (0.15)	-0.36892 (0.15)
	Bias	0.00462 (0.15)	0.00271 (0.15)	-0.00062 (0.15)	-0.00097 (0.15)	0.00943 (0.15)	0.01269 (0.15)
	Mean Squared Error	0.04478 (0.03)	0.04799 (0.03)	0.04476 (0.03)	0.04306 (0.03)	0.04485 (0.03)	0.04474 (0.03)
	Coverage, n (%)	942 (94.20)	937 (93.70)	945 (94.50)	952 (95.20)	939 (93.90)	951 (95.10)
	Power, n (%)	695 (69.50)	689 (68.90)	695 (69.50)	645 (64.50)	695 (69.50)	688 (68.80)
Two stage Cox IV	Effect Estimate	-0.36935 (0.15)	-0.35943 (0.15)	-0.36935 (0.15)	-0.34265 (0.15)	-0.36935 (0.15)	-0.36482 (0.15)
	Bias	0.00084 (0.15)	0.00755 (0.15)	-0.0044 (0.15)	0.00287 (0.15)	0.00565 (0.15)	0.01679 (0.15)
	Mean Squared Error	0.04381 (0.03)	0.04708 (0.03)	0.04383 (0.03)	0.04321 (0.03)	0.04384 (0.03)	0.04477 (0.03)
	Coverage, n (%)	952 (95.20)	946 (94.60)	951 (95.10)	955 (95.50)	951 (95.10)	954 (95.40)
	Power, n (%)	690 (69.00)	671 (67.10)	690 (69.00)	629 (62.90)	690 (69.00)	669 (66.90)

Table 6. Performance of IV methods in a scenario with a large treatment effect, strong confounding, moderate IV and low survival probability $S(5) = 0.3$ ($N = 10,000$). Model estimates are compared to the true marginal effect at 5 years follow-up. Results are summarised across the 1,000 simulations. Cox models are compared to the marginal hazard ratio whilst additive models are compared to the marginal hazard difference.

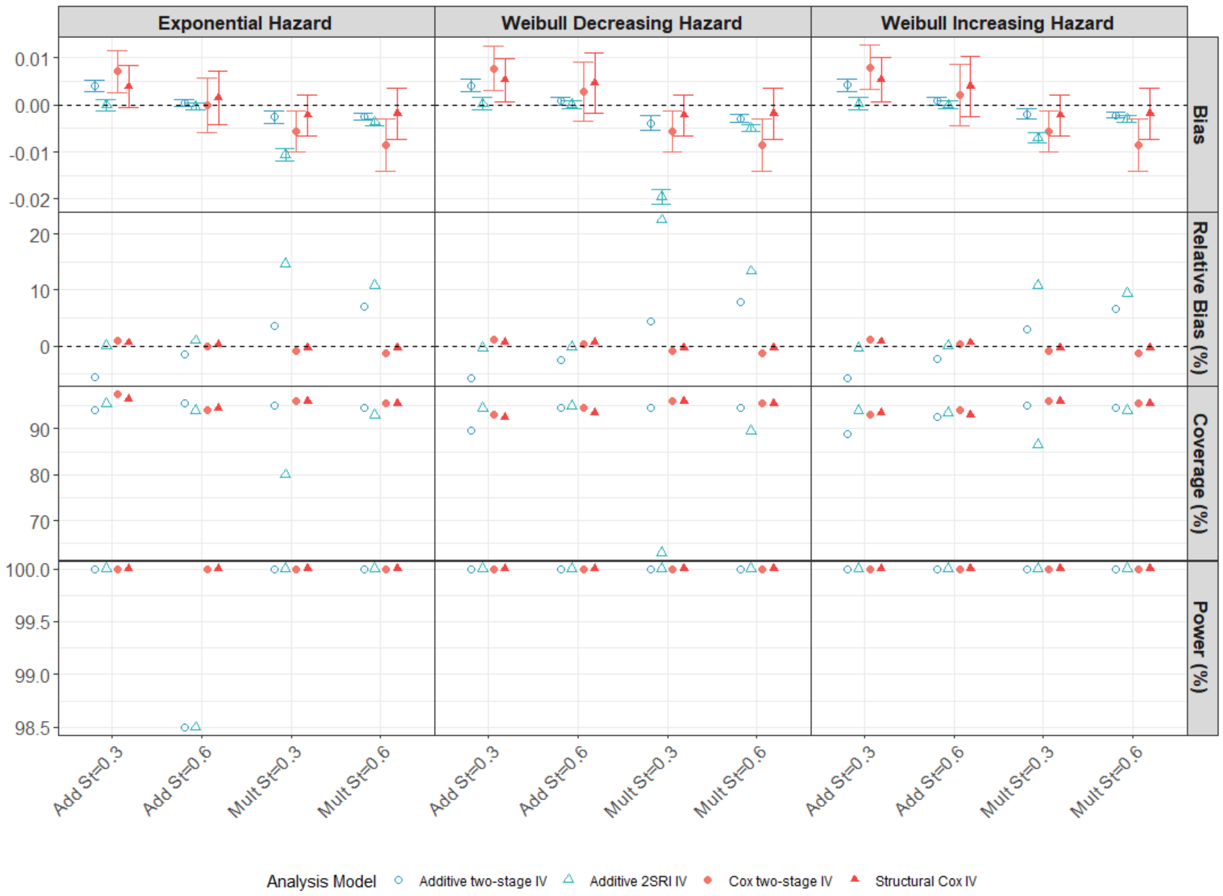

Figure 18. Performance of IV methods for a scenario with a large treatment effect, strong confounding and a moderate IV for $N = 100,000$. Low ($S(5) = 0.3$) and high ($S(5) = 0.6$) survival probabilities are plotted across the x-axis for both additive (Add) and multiplicative (Mult) DGMs. Each vertical panel presents a different baseline hazard distribution specified in the data generating model. Points represent the average across the 200 simulations. Error bars are 95% intervals using Monte-Carlo standard errors. Cox models are compared to the marginal hazard ratio whilst additive models are compared to the marginal hazard difference.

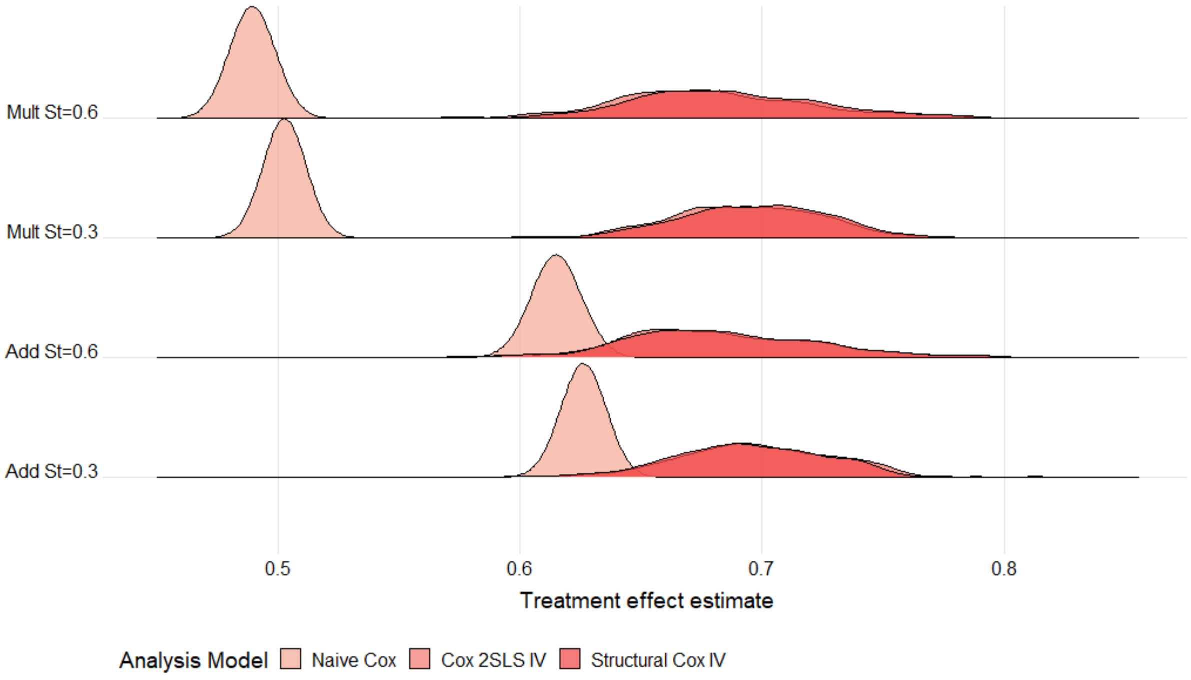

Figure 19. Ridgeline plot of treatment effect estimates for the Cox models across 200 datasets for a scenario with moderate IV, strong confounding and a large treatment effect under an exponential baseline hazard at 5 years follow-up and $N = 100,000$). The data generating mechanism is given on the y-axis: additive (Add) or multiplicative (Mult) DGM and high (0.6) or low (0.3) 5-year survival probability. The true hazard-ratios are: Mult S(5)=0.6 HR= 0.688; Mult S(5)=0.3 HR=0.699; Add S(5)=0.6 HR=0.683; Add S(5)=0.3 HR=0.691. Note, all three curves are plotted however the distributions for the two IV models mostly overlap.

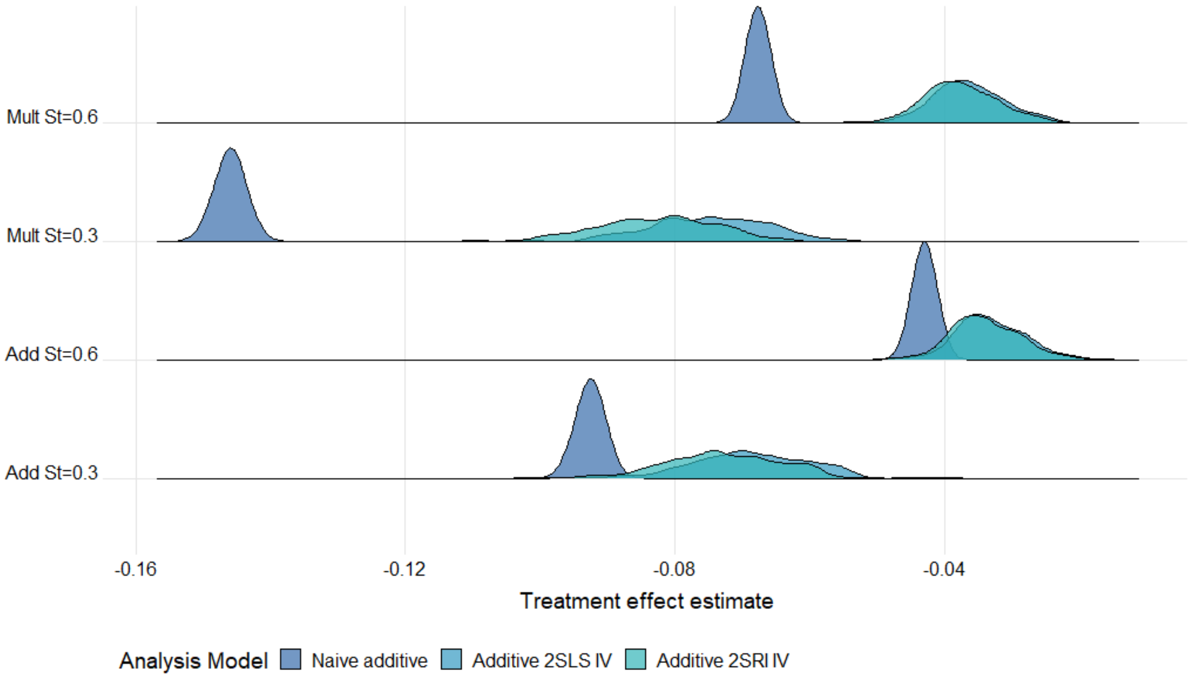

Figure 20. Ridgeline plot of treatment effect estimates for the additive models across 200 datasets for a scenario with moderate IV, strong confounding and a large treatment effect under an exponential baseline hazard at 5 years follow-up and $N = 100,000$). The data generating mechanism is given on the y-axis: additive (Add) or multiplicative (Mult) DGM and high (0.6) or low (0.3) 5-year survival probability. The true hazard-differences are: Mult S(5)=0.6 HD= -0.034; Mult S(5)=0.3 HD=-0.072; Add S(5)=0.6 HD=-0.034; Add S(5)=0.3 HD=-0.073. Note, all three curves are plotted however the distributions for the two IV models mostly overlap.

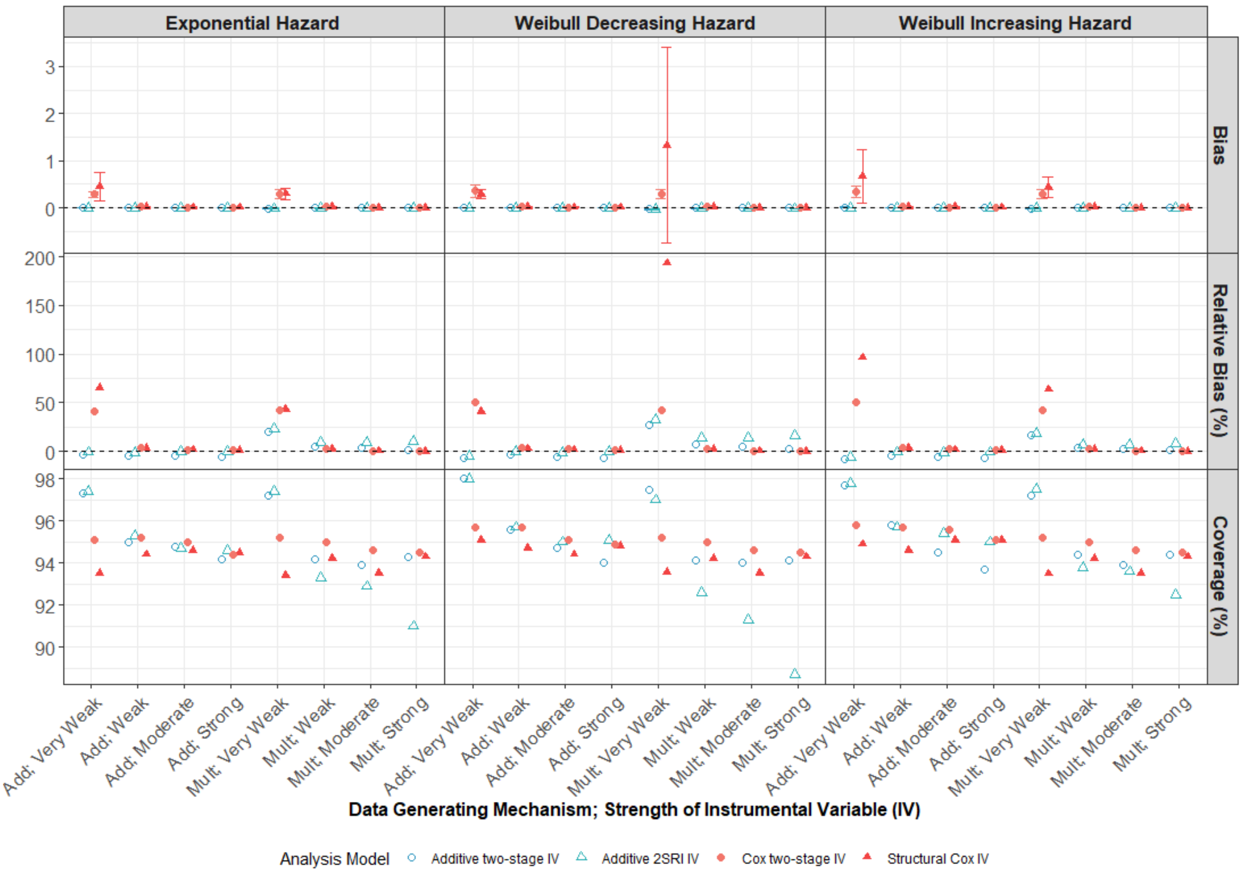

Figure 21. Performance of IV methods across different IV strengths compared to the marginal true parameter at 5 years follow-up ($N = 10,000$). Scenario with large treatment effect, moderate confounding and low survival probability $S(5) = 0.3$. Very weak, weak, moderate and strong instrument strengths are plotted across the x-axis for both additive (Add) and multiplicative (Mult) DGMs. Points represent the average across the 1,000 simulations. Error bars are 95% intervals using Monte-Carlo standard errors.

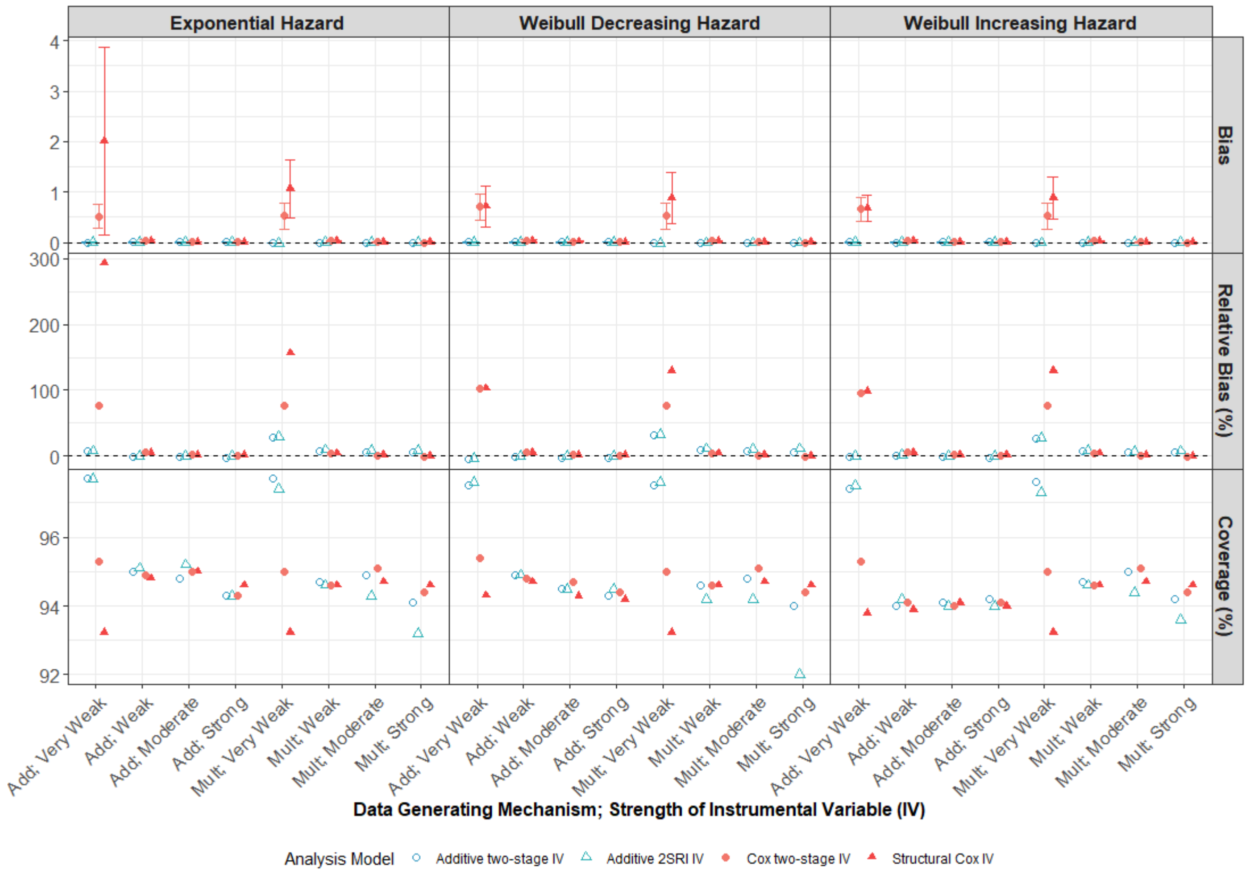

Figure 22. Performance of IV methods across different IV strengths compared to the marginal true parameter at 5 years follow-up for a scenario with large treatment effect, moderate confounding and high survival probability $S(5) = 0.6$ (N=10,000). Very weak, weak, moderate and strong instrument strengths are plotted across the x-axis for both additive (Add) and multiplicative (Mult) DGMs. Points represent the average across the 1,000 simulations. Error bars are 95% intervals using Monte-Carlo standard errors.

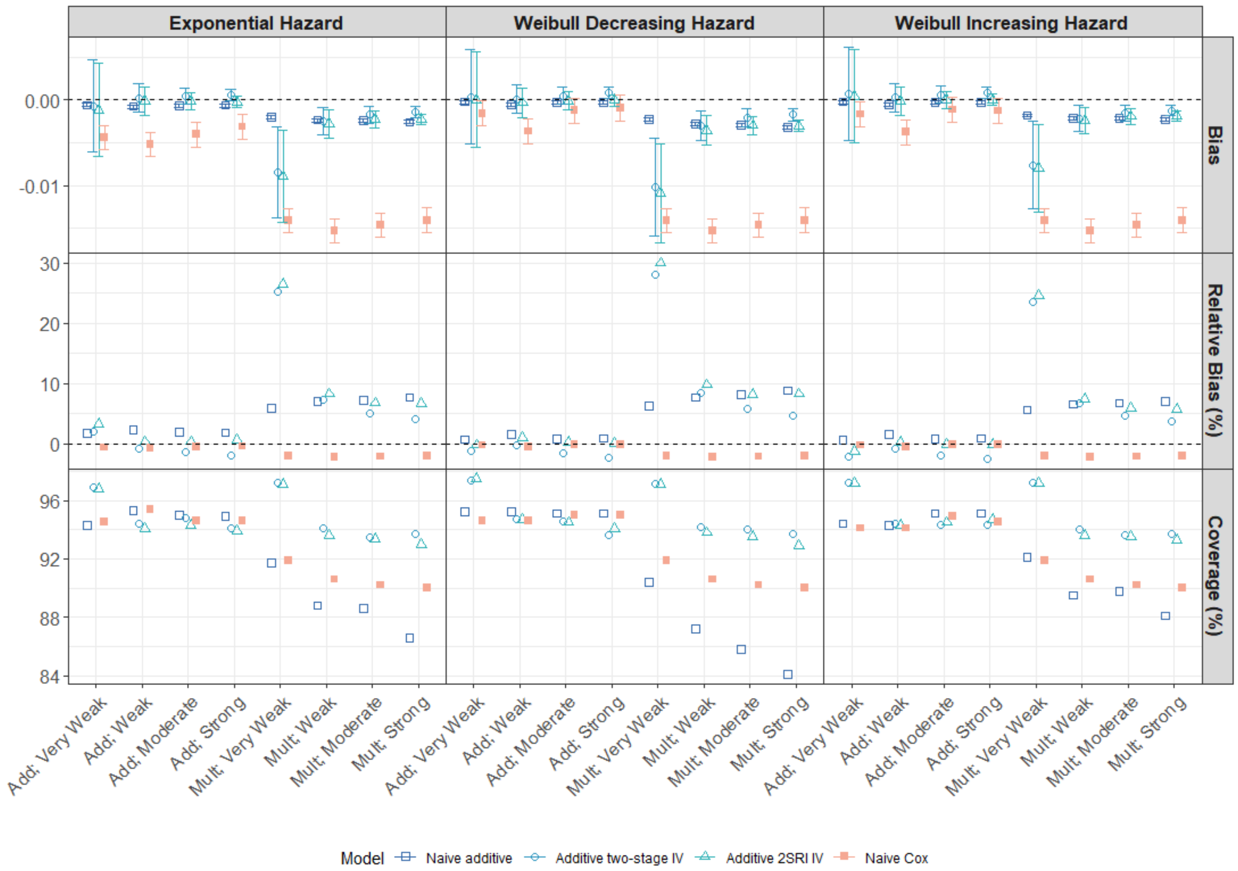

Figure 23. Performance of naïve and additive IV methods (Cox IV results excluded) across different IV strengths compared to the marginal true parameter at 5 years follow-up (N=10,000). Scenario with large treatment effect, *weak* confounding and low survival probability $S(5) = 0.3$. Very weak, weak, moderate and strong instrument strengths are plotted across the x-axis for both additive (Add) and multiplicative (Mult) DGMs. Points represent the average across the 1,000 simulations. Error bars are 95% intervals using Monte-Carlo standard errors.

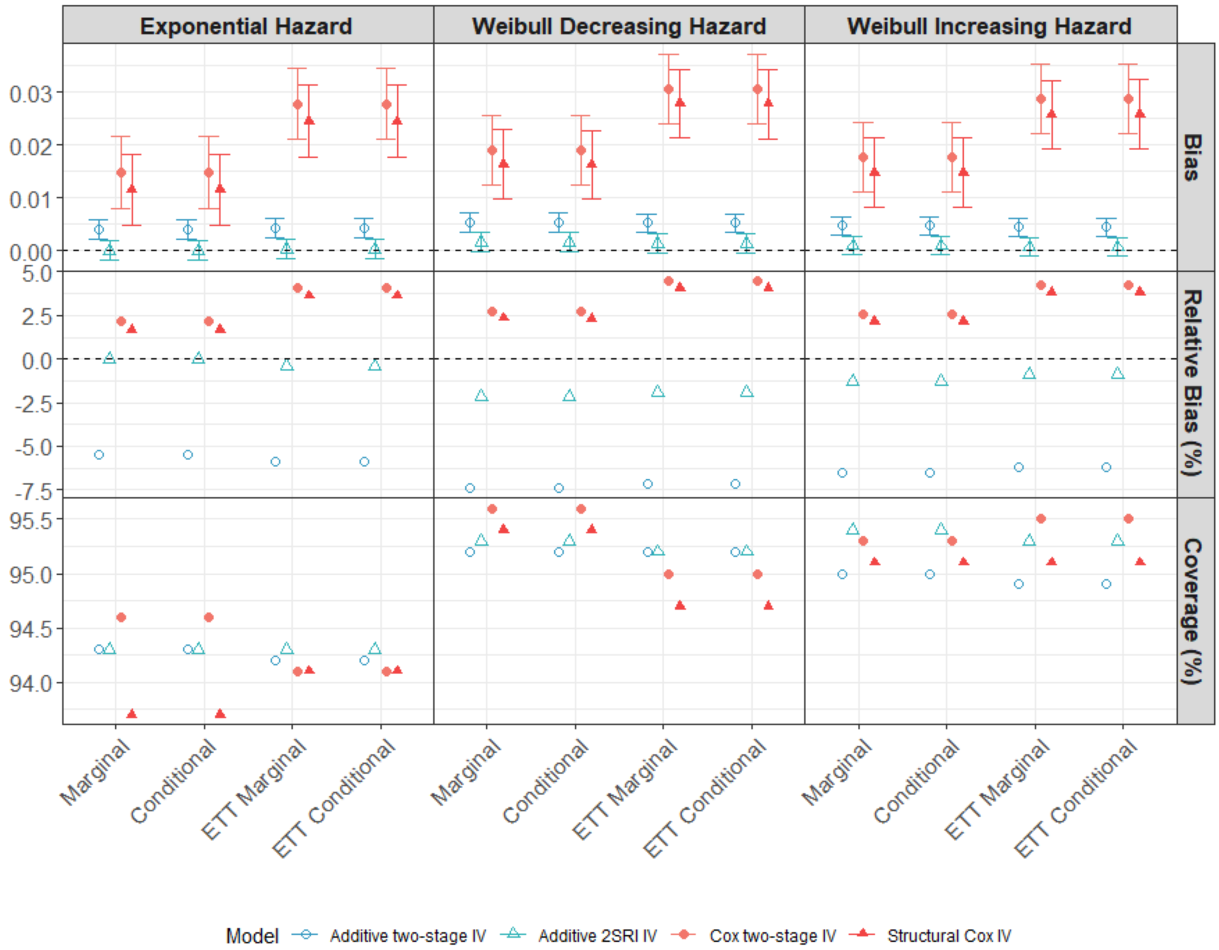

Figure 24. Performance of IV methods across different target estimands at 5 years follow-up for an additive DGM ($N = 10,000$). Scenario with large treatment effect, strong confounding, moderate IV and low survival probability $S(5) = 0.3$. Points represent the average across the 1,000 simulations. Error bars are 95% intervals using Monte-Carlo standard errors. Results for just the IV methods are presented here.

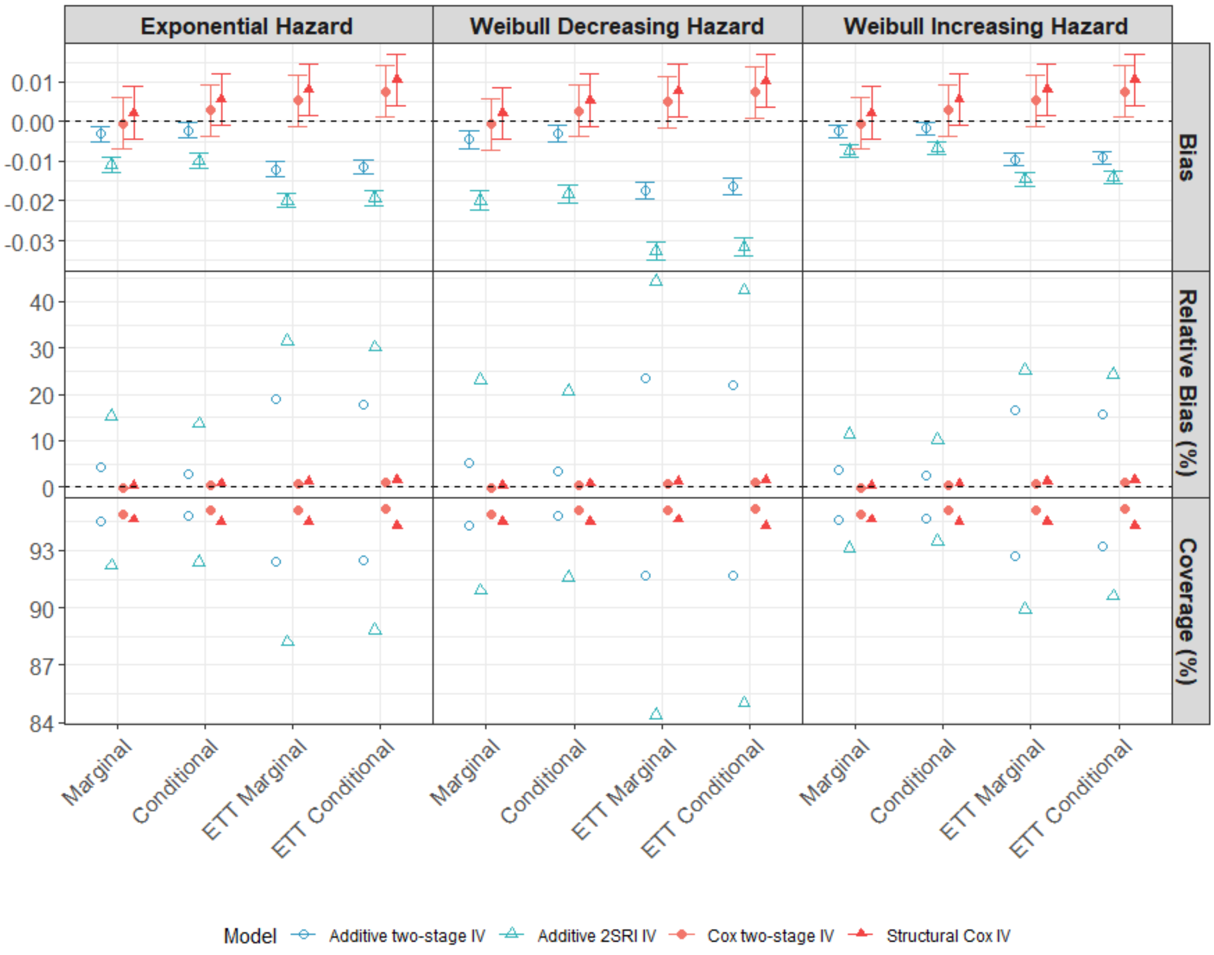

Figure 25. Performance of IV methods across different target estimands at 5 years follow-up for a multiplicative DGM ($N = 10,000$). Scenario with large treatment effect, strong confounding, moderate IV and low survival probability $S(5) = 0.3$. Points represent the average across the 1,000 simulations. Error bars are 95% intervals using Monte-Carlo standard errors. Results for just the IV methods are presented here.

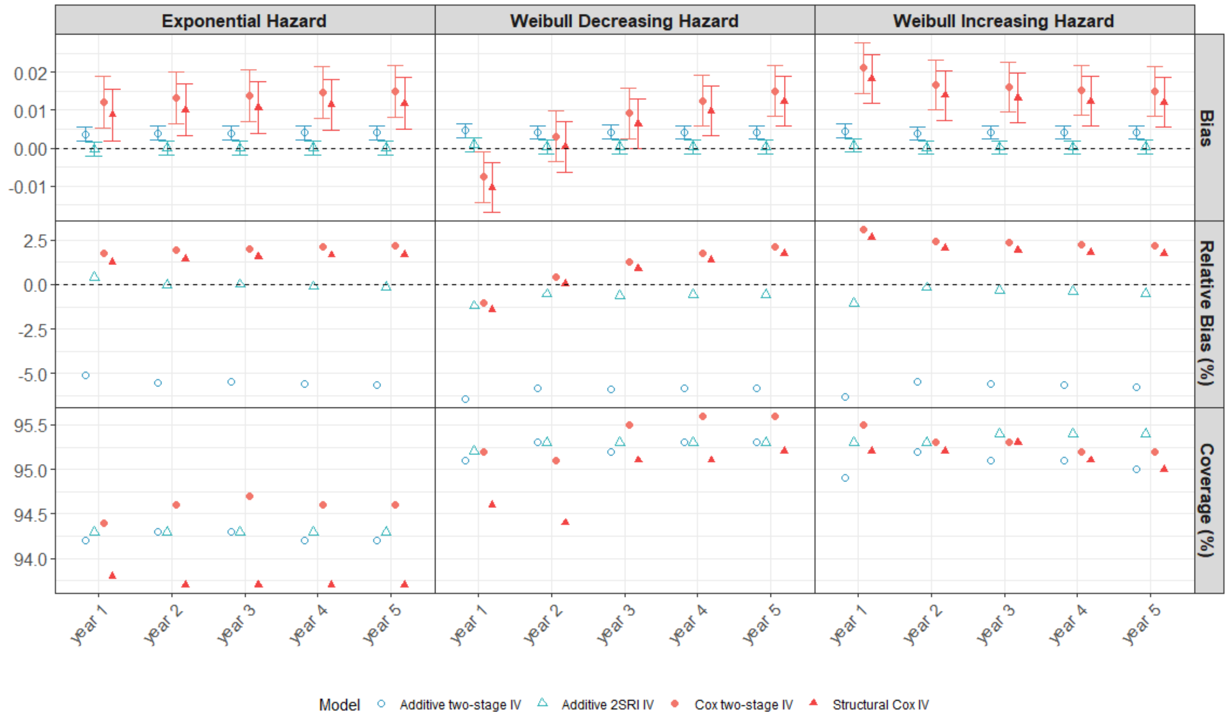

Figure 26. Performance of IV methods compared to the marginal true parameter at years $1, \ldots, 5$ of follow-up ($N = 10,000$). Estimates from additive methods were compared to the true hazard difference at each year. Estimates from Cox methods were compared to the true hazard ratio at each year. Scenario with an additive DGM, large treatment effect, strong confounding, moderate IV and low survival probability $S(5) = 0.3$. X-axis gives the year of follow up to which the estimates were compared. Points represent the average across the 1,000 simulations. Error bars are 95% intervals using Monte-Carlo standard errors.

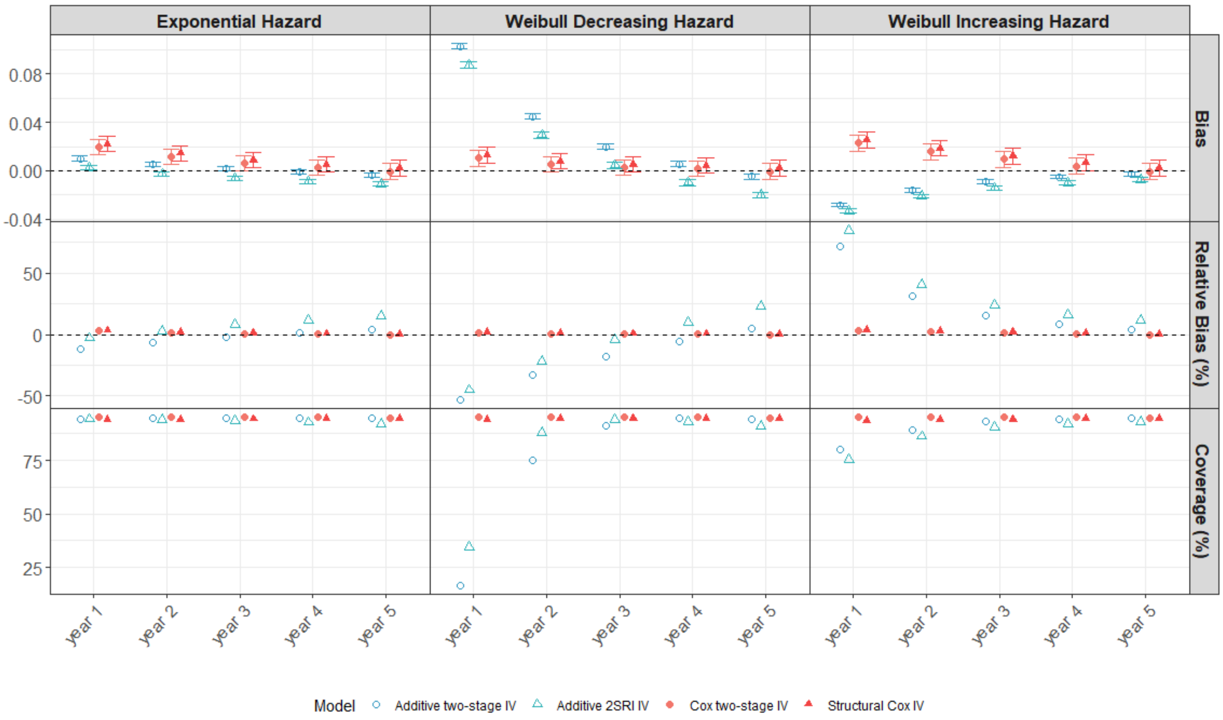

Figure 27. Performance of IV methods compared to the marginal true parameter at years $1, \dots, 5$ of follow-up $(N = 10,000)$. Estimates from additive methods were compared to the true hazard difference at each year. Estimates from Cox methods were compared to the true hazard ratio at each year. Scenario with a multiplicative DGM, large treatment effect, strong confounding, moderate IV and low survival probability $S(5) = 0.3$. X-axis gives the year of follow up to which the estimates were compared. Points represent the average across the 1,000 simulations. Error bars are 95% intervals using Monte-Carlo standard errors.

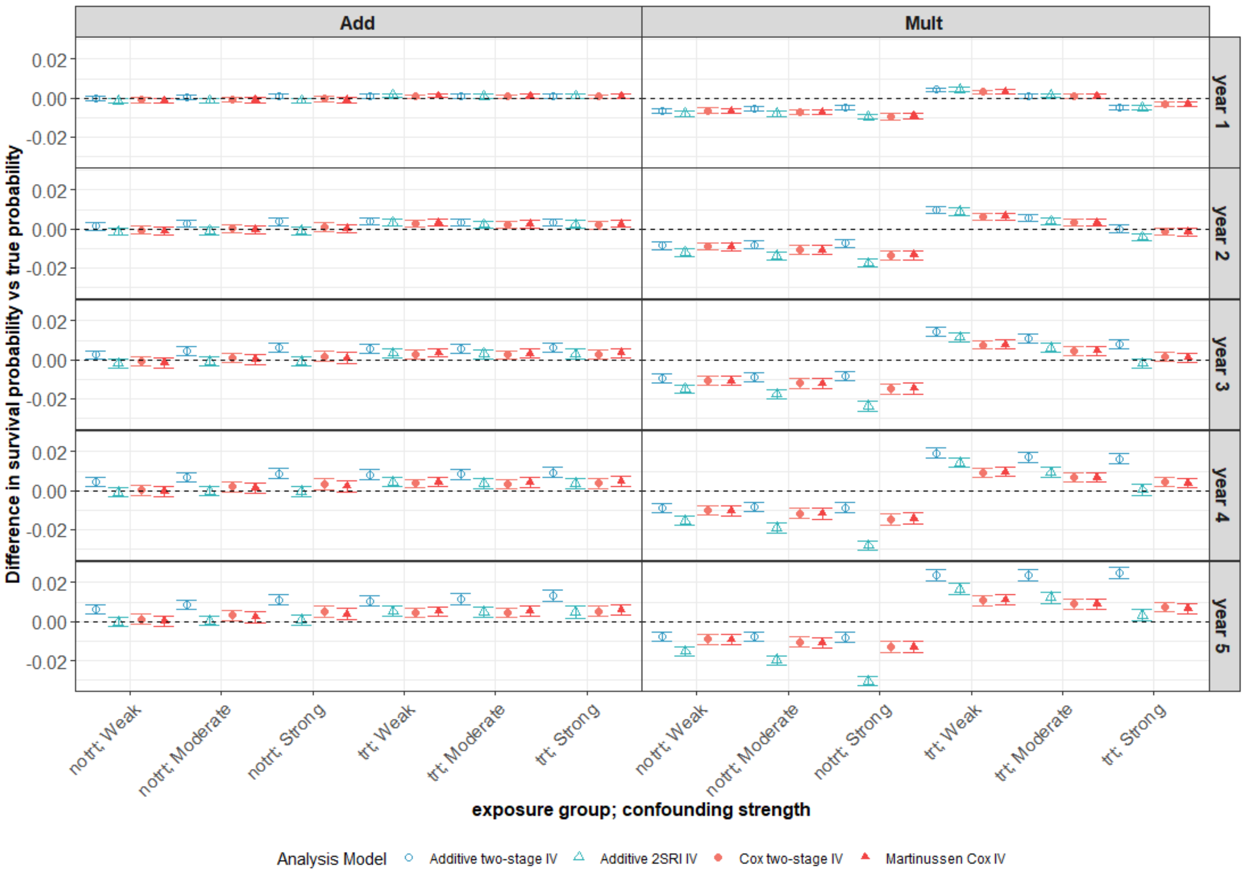

Figure 28. Difference between survival probability predictions and the true marginal survival probabilities for different confounding strengths. Scenario with large treatment effect under an exponential baseline hazard, weak IV and $S(5) = 0.3$ and $N = 10,000$. Weak, moderate and strong confounding strengths are plotted across the x-axis for both treated (trt) and untreated (notrt) exposure groups. Points represent the average across the 1,000 simulations. Error bars are 95% intervals using Monte-Carlo standard errors.

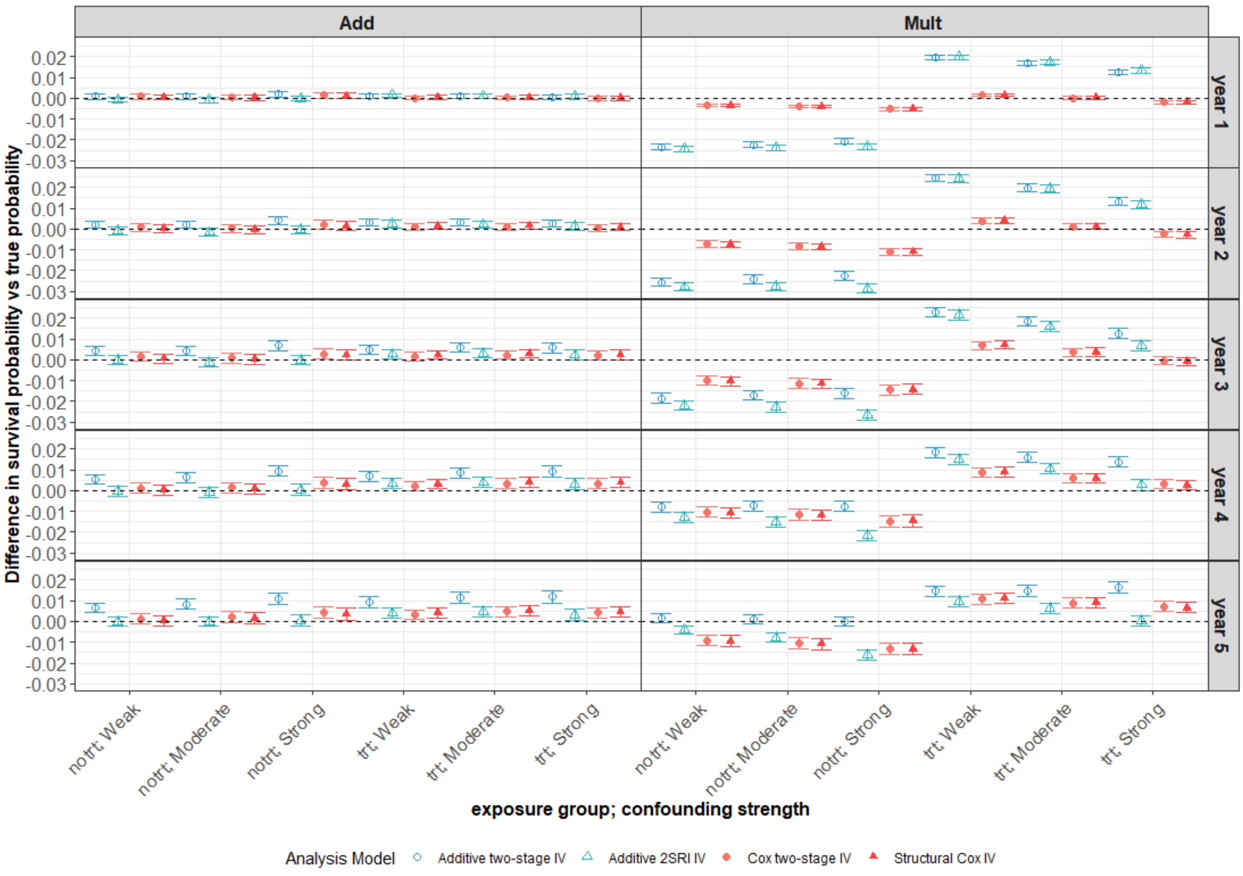

Figure 29. Difference between survival probability predictions and the true marginal survival probabilities for different confounding strengths. Scenario with large treatment effect under an increasing Weibull baseline hazard, weak IV and $S(5) = 0.3$ $(N = 10,000)$. Weak, moderate and strong confounding strengths are plotted across the x-axis for both treated (trt) and untreated (notrt) exposure groups. Points represent the average across the 1,000 simulations. Error bars are 95% intervals using Monte-Carlo standard errors.

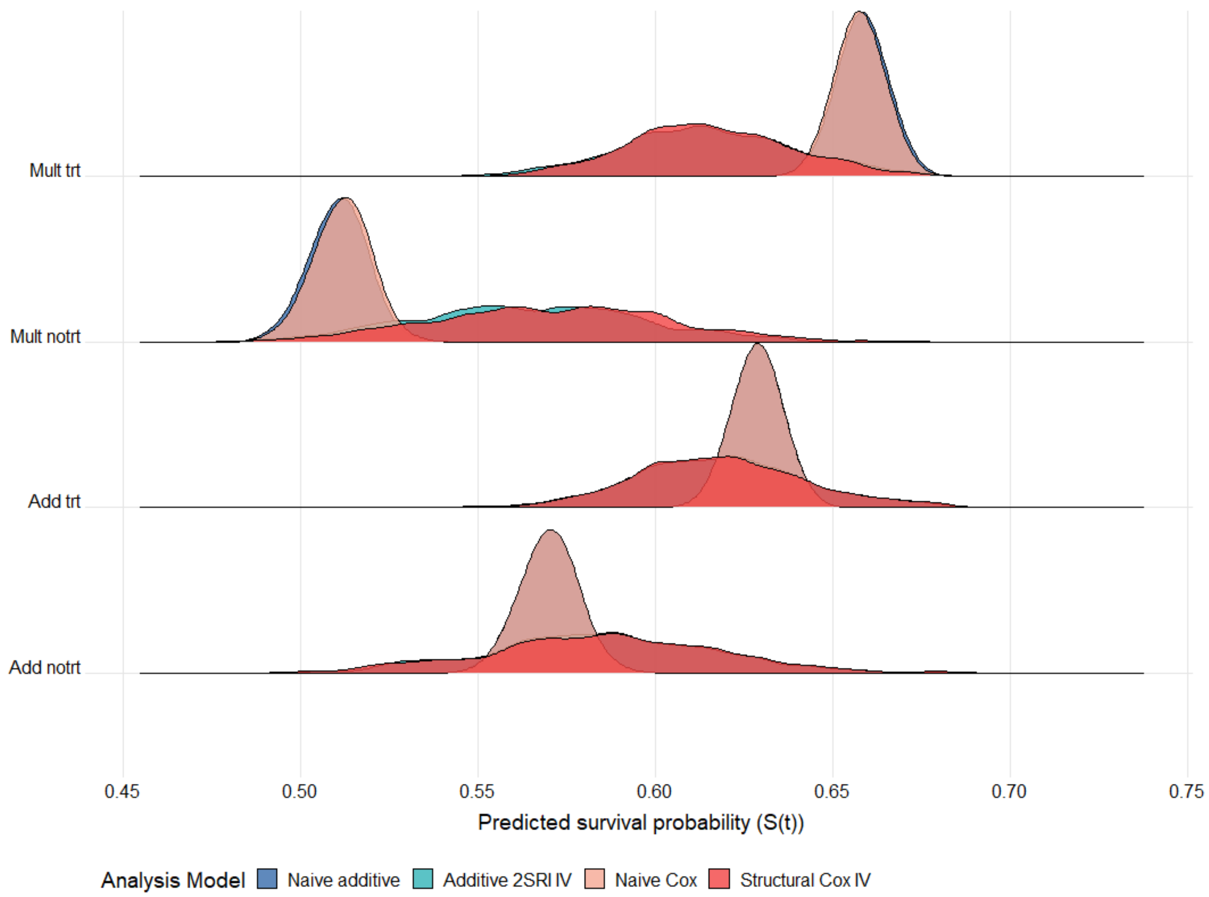

Figure 30. Ridgeline plot of survival probability predictions at 5 years follow up across $1,000$ datasets for the naïve and 2SRI additive and structural Cox IV methods. Scenario with a moderate IV, strong confounding and a large treatment effect under an exponential baseline hazard and $S(5) = 0.6$ $(N = 10,000)$. X-axis gives the survival probability predictions under each method. The data generating mechanism is given on the y-axis: additive (Add) or multiplicative (Mult) DGM, along with the exposure arm: treated (trt) or untreated (notrt). True survival probabilities: Mult trt = 0.689, notrt = 0.582; Add trt = 0.696, notrt = 0.589.

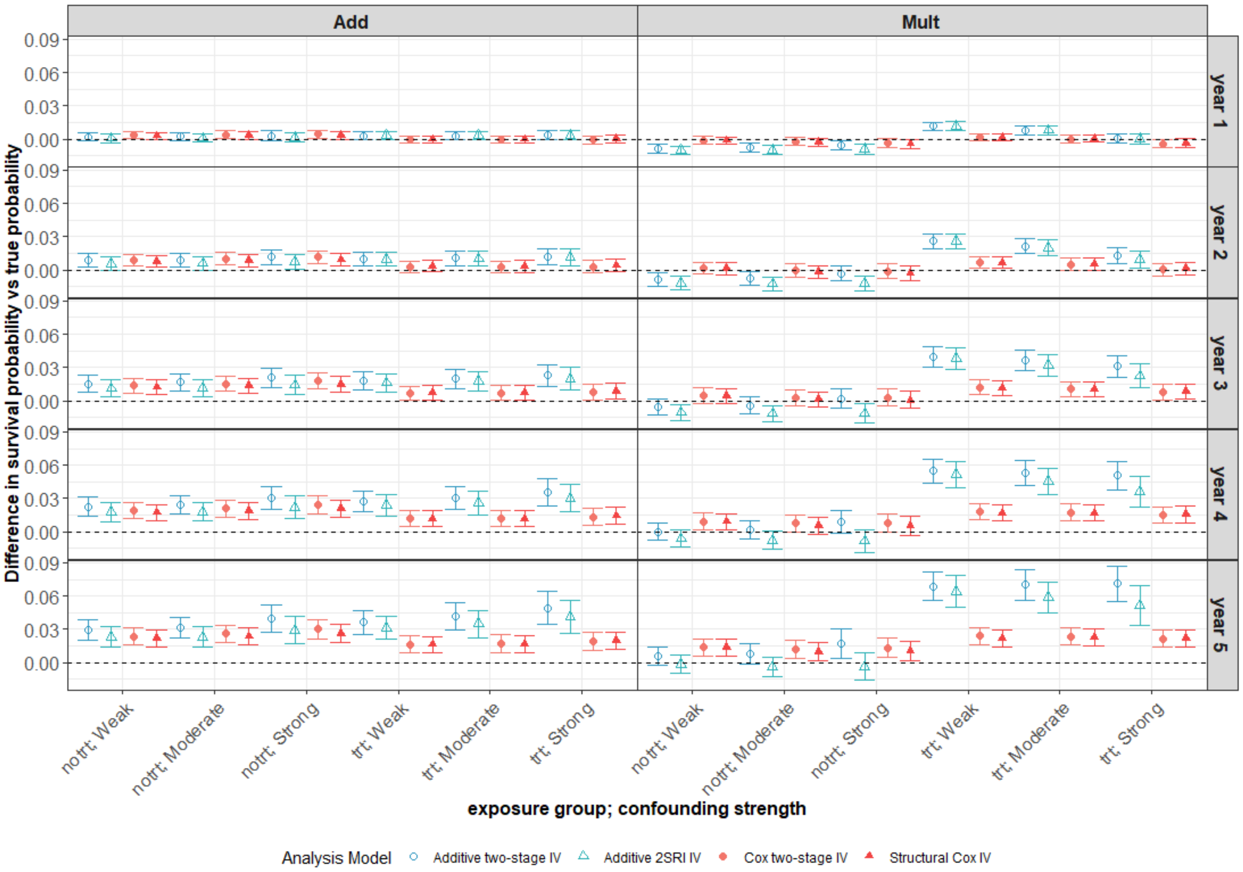

Figure 31. Difference between survival probability predictions and the true marginal survival probabilities for different confounding strengths. Scenario with large treatment effect under an exponential baseline hazard, very weak IV and $S(5) = 0.3$ $(N = 10,000)$. Weak, moderate and strong confounding strengths are plotted across the x-axis for both treated (trt) and untreated (notrt) exposure groups. Points represent the average across the $1,000$ simulations. Error bars are 95% intervals using Monte-Carlo standard errors.

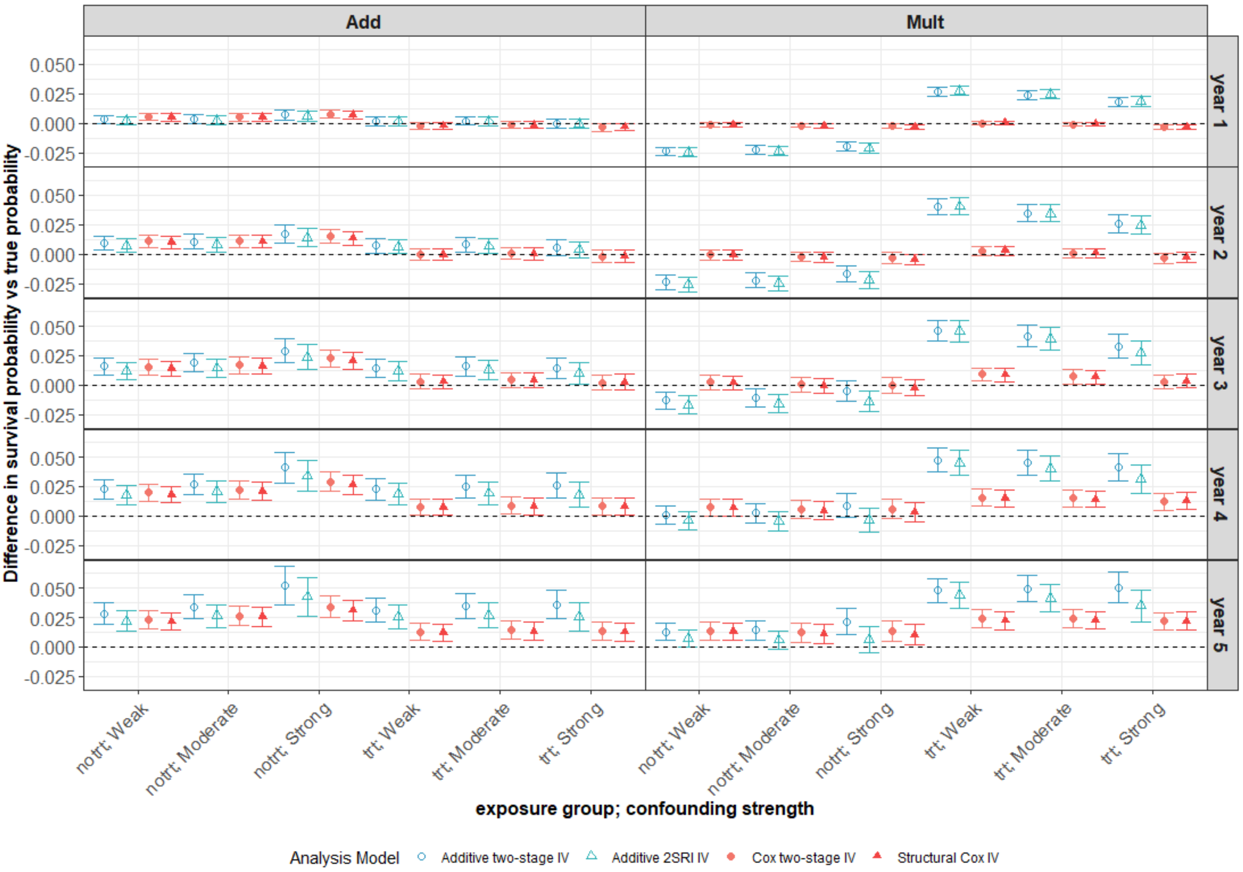

Figure 32. Difference between survival probability predictions and the true marginal survival probabilities for different confounding strengths. Scenario with large treatment effect under an increasing Weibull baseline hazard, very weak IV and $S(5) = 0.3$ $(N = 10,000)$. Weak, moderate and strong confounding strengths are plotted across the x-axis for both treated (trt) and untreated (notrt) exposure groups. Points represent the average across the $1,000$ simulations. Error bars are 95% intervals using Monte-Carlo standard errors.

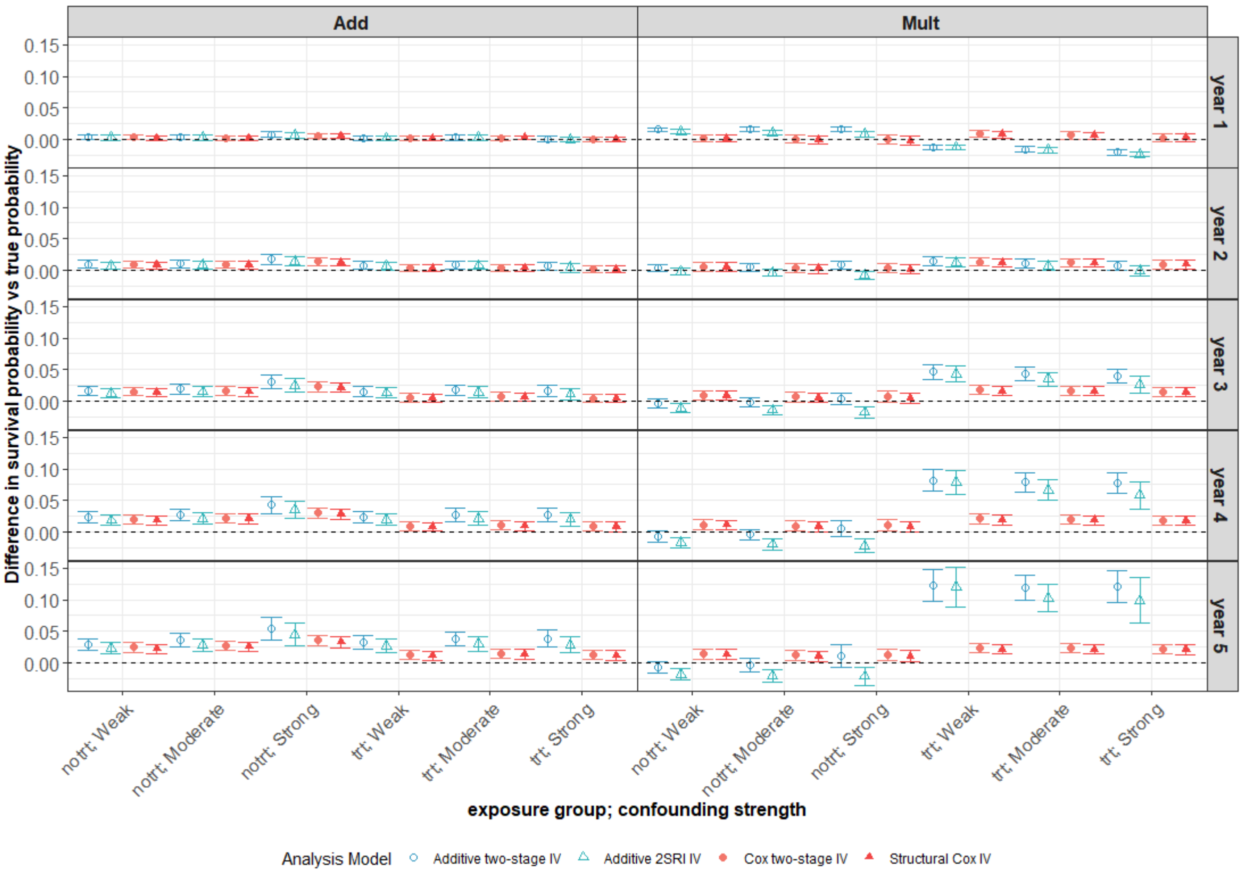

Figure 33. Difference between survival probability predictions and the true marginal survival probabilities for different confounding strengths. Scenario with large treatment effect and very weak IV under a decreasing Weibull baseline hazard with $S(5) = 0.3$ $(N = 10,000)$. Weak, moderate and strong confounding strengths are plotted across the x-axis for both treated (trt) and untreated (notrt) exposure groups. Points represent the average across the $1,000$ simulations. Error bars are 95% intervals using Monte-Carlo standard errors.

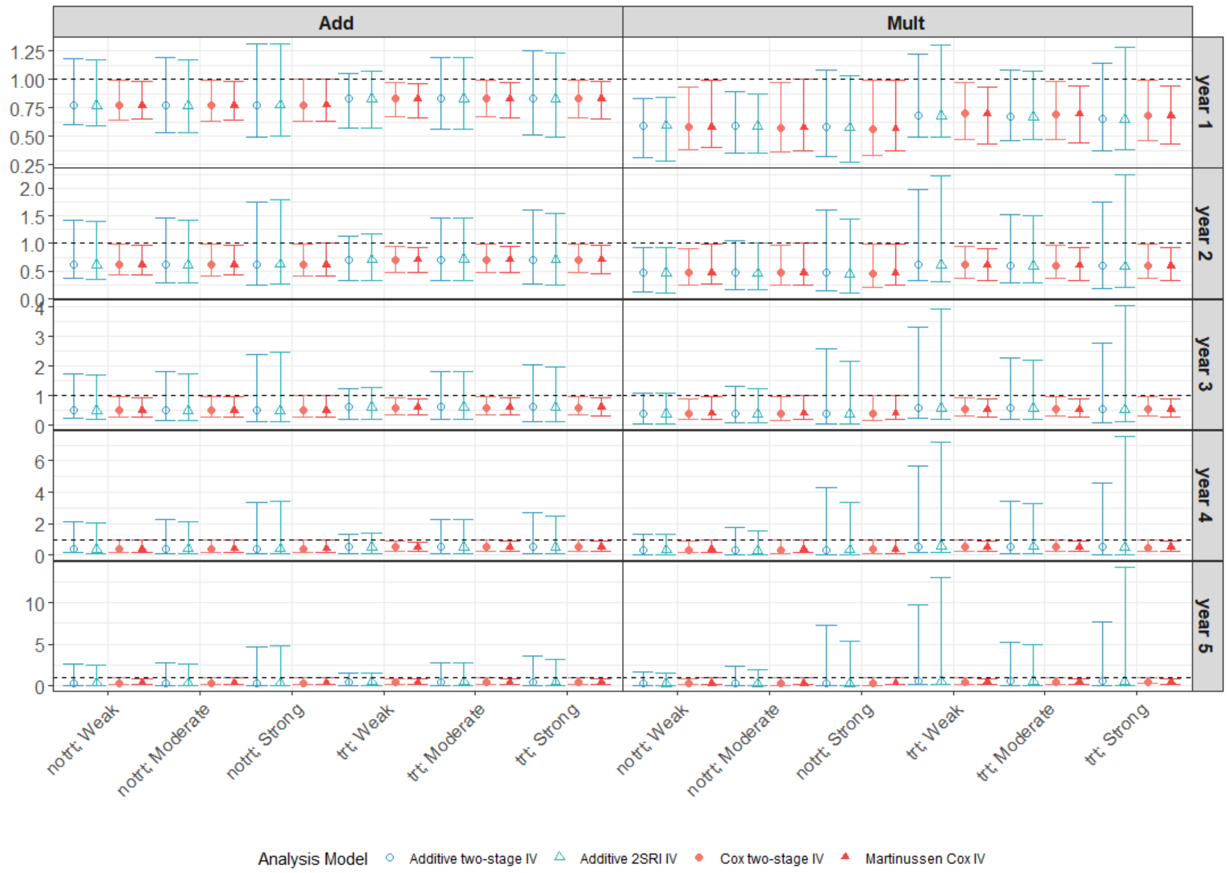

Figure 34. Minimum and maximum survival predictions for different strengths of confounding. Weak, moderate and strong confounding strengths are plotted across the x-axis for both treated (trt) and untreated (notrt) exposure groups. Scenario with large treatment effect, decreasing Weibull baseline hazard, very weak IV and $S(5) = 0.3$ $(N = 10,000)$. Points represent the average survival probability across the $1,000$ simulations. Error bars give the minimum and maximum observed survival probability across the $1,000$ simulations.

Data example results

These results are from the CPRD data example.

Results of the naïve regression models for Cox and additive regression.

	Naïve Additive		Naïve Cox		
	Coefficient (SE)	p-value	Log(HR) (SE)	p-value	HR
Exposure					
Exposed (statin)	0.0099 (0.0001)	<0.001	1.518 (0.015)	<0.001	4.563
Hypertensive					
Yes	0.0020 (0.0001)	<0.001	0.308 (0.015)	<0.001	1.360
Smoking category					
Ex-smoker	0.0010 (0.0001)	<0.001	0.185 (0.015)	<0.001	1.203
Current smoker	0.0024 (0.0001)	<0.001	0.378 (0.017)	<0.001	1.460
Ethnicity					
Non-white	0.0057 (0.0003)	<0.001	0.793 (0.028)	<0.001	2.211
BMI	0.0008 (1.1e-05)	<0.001	0.112 (0.001)	<0.001	1.119
Age	2.8e-05 (3.7e-06)	<0.001	0.009 (0.000)	<0.001	1.009
Gender					
Female	-0.0011 (0.0001)	<0.001	-0.294 (0.014)	<0.001	0.745

Table 7. Naïve additive and Cox regression models of T2DM on statin treatment adjusted for clinical covariates

Table showing the relationship between the instrumental variable (CPRD risk score) and exposure (statin treatment). Both a logistic regression and linear regression model are presented.

	Linear IV model		Logistic IV model		
	Coefficient (SE)	p-value	Log OR (SE)	p-value	OR
Intercept	0.045 (0.003)	<0.001	-2.136 (0.016)	<0.001	0.118
CVD risk					
High (>=20%)	0.283 (0.002)	<0.001	1.290 (0.011)	<0.001	3.632
Hypertensive					
Yes	0.277 (0.001)	<0.001	1.261 (0.006)	<0.001	3.528
Smoking category					
Ex-smoker	0.030 (0.001)	<0.001	0.015 (0.006)	<0.001	1.164
Current smoker	0.079 (0.001)	<0.001	0.396 (0.007)	<0.001	1.486
Ethnicity					
Non-white	0.034 (0.003)	<0.001	0.017 (0.014)	<0.001	1.187
BMI	0.006 (0.000)	<0.001	0.027 (0.001)	<0.001	1.027

Table 8. Linear and logistic regression models for exposure (statins) on the instrumental variable (CVD risk); OR = odds ratio.

Results from the instrumental variables models for additive and Cox regression.

	Log(HR) (SE)	p-value	HR
Structural Cox IV model	1.933 (0.016)	<0.001	6.910
Two-stage Cox IV	0.926 (0.087)	<0.001	2.525
	Coefficient (SE)	p-value	
Two-stage additive IV	0.0114(0.0008)	<0.001	
Two-stage RI additive IV	0.0129 (0.0011)	<0.001	

Table 9. Instrumental variables models of T2DM on exposure (statins).
